# Design and in vitro realization of carbon-conserving photorespiration

**DOI:** 10.1073/pnas.1812605115

**Published:** 2018-11-20

**Authors:** Devin L. Trudeau, Christian Edlich-Muth, Jan Zarzycki, Marieke Scheffen, Moshe Goldsmith, Olga Khersonsky, Ziv Avizemer, Sarel J. Fleishman, Charles A. R. Cotton, Tobias J. Erb, Dan S. Tawfik, Arren Bar-Even

**Affiliations:** ^a^Department of Biomolecular Sciences, Weizmann Institute of Science, 7610001 Rehovot, Israel;; ^b^Max Planck Institute of Molecular Plant Physiology, 14476 Potsdam-Golm, Germany;; ^c^Max Planck Institute for Terrestrial Microbiology, D-35043 Marburg, Germany

**Keywords:** carbon fixation, computational modeling, kinetic modeling, metabolic engineering, enzyme engineering

## Abstract

Photorespiration recycles ribulose-1,5-bisphosphate carboxylase/oxygenase (Rubisco) oxygenation product, 2-phosphoglycolate, back into the Calvin Cycle. Natural photorespiration, however, limits agricultural productivity by dissipating energy and releasing CO_2_. Several photorespiration bypasses have been previously suggested but were limited to existing enzymes and pathways that release CO_2_. Here, we harness the power of enzyme and metabolic engineering to establish synthetic routes that bypass photorespiration without CO_2_ release. By defining specific reaction rules, we systematically identified promising routes that assimilate 2-phosphoglycolate into the Calvin Cycle without carbon loss. We further developed a kinetic–stoichiometric model that indicates that the identified synthetic shunts could potentially enhance carbon fixation rate across the physiological range of irradiation and CO_2_, even if most of their enzymes operate at a tenth of Rubisco’s maximal carboxylation activity. Glycolate reduction to glycolaldehyde is essential for several of the synthetic shunts but is not known to occur naturally. We, therefore, used computational design and directed evolution to establish this activity in two sequential reactions. An acetyl-CoA synthetase was engineered for higher stability and glycolyl-CoA synthesis. A propionyl-CoA reductase was engineered for higher selectivity for glycolyl-CoA and for use of NADPH over NAD^+^, thereby favoring reduction over oxidation. The engineered glycolate reduction module was then combined with downstream condensation and assimilation of glycolaldehyde to ribulose 1,5-bisphosphate, thus providing proof of principle for a carbon-conserving photorespiration pathway.

## Significance

Photorespiration limits plant carbon fixation by releasing CO_2_ and using cellular resources to recycle the product of ribulose-1,5-bisphosphate carboxylase/oxygenase (Rubisco) oxygenation, 2-phosphoglycolate. We systematically designed synthetic photorespiration bypasses that combine existing and new-to-nature enzymatic activities and that do not release CO_2_. Our computational model shows that these bypasses could enhance carbon fixation rate under a range of physiological conditions. To realize the designed bypasses, a glycolate reduction module, which does not exist in nature, is needed to be engineered. By reshaping the substrate and cofactor specificity of two natural enzymes, we established glycolate reduction to glycolaldehyde. With the addition of three natural enzymes, we observed recycling of glycolate to the key Calvin Cycle intermediate ribulose 1,5-bisphosphate with no carbon loss.

Current annual rates of agricultural yield improvement fall far short of the estimated 2.4% rate required to feed the global population by 2050 (1). To address humanity’s need in the long run, we will have to find a way to increase the efficiency with which solar energy is converted into biomass (2, 3). Such an improvement could result from optimizing the light reactions (4), CO_2_ diffusion and concentration (5), activity of ribulose-1,5-bisphosphate carboxylase/oxygenase (Rubisco) (6), or the regeneration of ribulose 1,5-bisphosphate (RuBP) (7). Among these processes, photorespiration was identified as a primary target for engineering toward increased carbon fixation rate and yield (2, 3, 8, 9).

Photorespiration assimilates 2-phosphoglycolate (2PG)—the product of Rubisco’s oxygenation reaction (8, 9). This process channels a very high metabolic flux and is vital to avoid the inhibitory effect of 2PG (10, 11) and to recycle carbon back into the Calvin Cycle. However, plant photorespiration is inefficient, as it dissipates energy and reducing power and most importantly, releases carbon—one CO_2_ per two glycolate molecules recycled. This CO_2_ release necessitates more turns of the Calvin Cycle, thus lowering the carbon fixation rate and further increasing the energy demand per carbon fixed. It is estimated that photorespiration leads to loss of up to 30% of the carbon fixed via photosynthesis (3).

Several photorespiration bypasses have been proposed and at least partially tested (12–15). However, most of these still generally follow the carbon stoichiometry of native photorespiration, releasing one CO_2_ molecule per two glycolate molecules recycled. Hence, while offering higher energetic efficiency, these alternative routes fall short of addressing the main problem of photorespiration. CO_2_-neutral and CO_2_-positive photorespiration shunts have previously been suggested (3, 16, 17) but were mostly limited to the production of intermediates that are not directly reassimilated to the Calvin Cycle [e.g., pyruvate (16) and acetyl-CoA (17)]. Moreover, a comprehensive analysis that uncovers the optimal structure of such pathways is still missing.

In this study, we systematically search and analyze photorespiration bypass routes that do not release CO_2_. By considering specific biochemical reaction rules, we identified all pathway structures that could recycle 2PG into the Calvin Cycle without loss of carbon. These pathways harbor metabolites and reactions that do not necessarily exist in nature but could plausibly be realized by engineering natural enzymes. By manually analyzing the pathway candidates, we uncovered those with promising physicochemical properties. We further developed a computational model showing that the carbon-conserving pathways can significantly boost plant productivity. We then demonstrate the in vitro implementation of one of these synthetic routes. Specifically, we describe the successful engineering of two enzymes that together catalyze a new-to-nature transformation: glycolate reduction to glycolaldehyde. An acetyl-CoA synthetase (ACS) was engineered toward higher stability and increased catalytic efficiency with glycolate, and an NADH-dependent propionyl-CoA reductase was engineered to use NADPH and for >10-fold increased selectivity for glycolyl-CoA over acetyl-CoA. These two engineered enzymes were subsequently combined with three existing enzymes to convert glycolate to RuBP, thus providing a proof of principle of a carbon-conserving photorespiration bypass.

## Results

### Systematic Search for Promising Synthetic Photorespiration Shunts That Do Not Release CO_2_.

We started by creating a set of generalized biochemical reaction rules: for example, reduction of a carbonyl to hydroxycarbon, activation of an acid to a phosphoanhydride or acyl-CoA, or aldol cleavage/condensation. Our set of reaction rules does not include reactions in which CO_2_ is released or reactions that are known to be oxygen sensitive (e.g., 2-oxoacid synthases). We further excluded phosphatase and acyl-CoA hydrolase reactions (other than 2PG phosphatase) as well as O_2_-dependent or quinone-dependent dehydrogenase reactions to avoid the unnecessary dissipation of energy and reducing power. We also established a set of rules defining which types of compounds are not permitted: for example, molecules with two carbonyl groups adjacent to one another (highly reactive) or molecules harboring both a phosphate and a CoA group (unprecedented in nature). All reaction and metabolite rules can be found in *SI Appendix*, section 1.

We then used the structure of 2PG as a starting point and explored all compounds that can be sequentially derived from it using the reaction rules and avoiding forbidden compounds. As our computational tool ignores the chirality of compounds, it identifies metabolic architectures rather than actual metabolic routes (that is, each metabolic architecture corresponds to several possible pathways according to the chirality of the metabolites). The metabolic architectures that we have identified are shown in *SI Appendix*.

Next, we analyzed each of the metabolic architectures to assess engineering feasibility. We applied a thermodynamic analysis to discard metabolic architectures that are thermodynamically limited (i.e., having maximum–minimum driving force lower than 1 kJ/mol) (18). We then performed a comprehensive literature review to identify the exact pathways that can be derived from each metabolic architecture (that is, which enantiomers could be produced with known enzymes and enzyme mechanisms). The pathways were further prioritized according to the following criteria: (*i*) small number of novel reactions [i.e., reactions not known to be catalyzed by an enzyme, and thus, they require enzyme engineering (one to three reactions)], (*ii*) known candidate promiscuous enzymes that could be engineered to catalyze the missing reactions, and (*iii*) limited overlap with endogenous metabolism, especially avoiding metabolic bridges between metabolic pathways that are naturally disconnected in the chloroplast (e.g., upper and lower glycolysis) (16, 19).

**Fig. 1.**
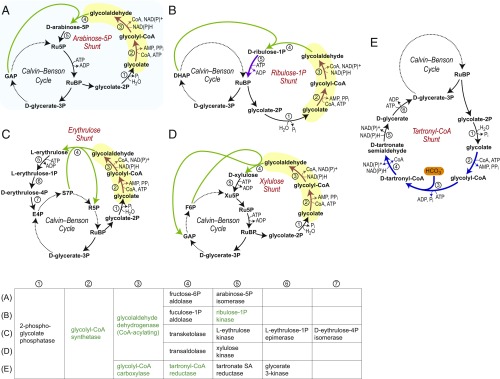
Promising photorespiration bypass routes. The first four routes (*A*–*D*) are carbon-neutral bypasses in which glycolate is reduced to glycolaldehyde (shown by brown arrows and yellow shading) followed by different glycolaldehyde-condensing reactions (in green). (*A*) Ar5P shunt, the synthetic pathway demonstrated in this study. (*B*) Ru1P shunt (Ru1P 5-kinase, which demands enzyme engineering, is shown in purple). (*C*) Erythrulose shunt. (*D*) Xylulose shunt. (*E*) The fifth route, a TrCoA shunt, is a carbon-positive bypass that demands the engineering of three enzymes (shown in blue). Enzyme names are given in the table; dark-green represents new-to-nature catalytic activities.

We identified two groups of promising synthetic photorespiration bypasses. The first group consists of carbon-neutral pathways in which glycolate is reduced to glycolaldehyde. Glycolaldehyde then undergoes aldol/ketol condensation with a sugar phosphate from the Calvin Cycle to generate a longer-chain sugar (phosphate) that is assimilated back into the cycle. Fig. 1 *A*–*D* presents the four most promising photorespiration bypasses that belong to this group. These synthetic pathways share a “glycolate reduction module” composed of two novel reactions (brown arrows and yellow shading in Fig. 1): (*i*) glycolate activation with CoA, which could be engineered starting from a promiscuous CoA-transferase (20) or an AMP- or ADP-forming CoA ligase (21), and (*ii*) glycolyl-CoA reduction to glycolaldehyde, which could be established from a promiscuous CoA-acylating aldehyde dehydrogenase (22). While carboxylic acid reductase can potentially catalyze glycolate reduction directly (23), its relative complexity makes its engineering more challenging.

Notably, the glycolaldehyde-condensing reactions (green arrows in Fig. 1) that participate in the bypass routes can be supported by the promiscuous action of natural enzymes: fuculose 1-phosphate aldolase (24), fructose 6-phosphate aldolase (25), transketolase (26), and transaldolase (27). The ribulose 1-phosphate (Ru1P) shunt, however, requires the engineering of another novel reaction (purple arrows in Fig. 1): Ru1P 5-kinase, which could, in principle, be engineered from a promiscuous ribulose kinase (28).

As an alternative pathway, the tartronyl-CoA (TrCoA) shunt (Fig. 1*E*) is carbon positive (that is, it relies on a carboxylation step, thereby directly supporting the activity of the Calvin Cycle). The TrCoA shunt requires the engineering of three novel reactions (blue arrows in Fig. 1): (*i*) glycolate activation with CoA as discussed above; (*ii*) carboxylation of glycoyl-CoA to TrCoA, which could be engineered from a promiscuous biotin-dependent carboxylase (29); and (*iii*) TrCoA reductase, which could be established starting from a promiscuous CoA-acylating aldehyde dehydrogenase. We note that glycolyl-CoA carboxylation would not be dependent on the low-concentration CO_2_ but would rather tap into the much larger pool of bicarbonate, the concentration of which is orders of magnitude higher than that of CO_2_ in the alkaline stroma.

### Synthetic Photorespiration Shunts Are Expected to Support Higher Carbon Fixation Rate.

**Fig. 2.**
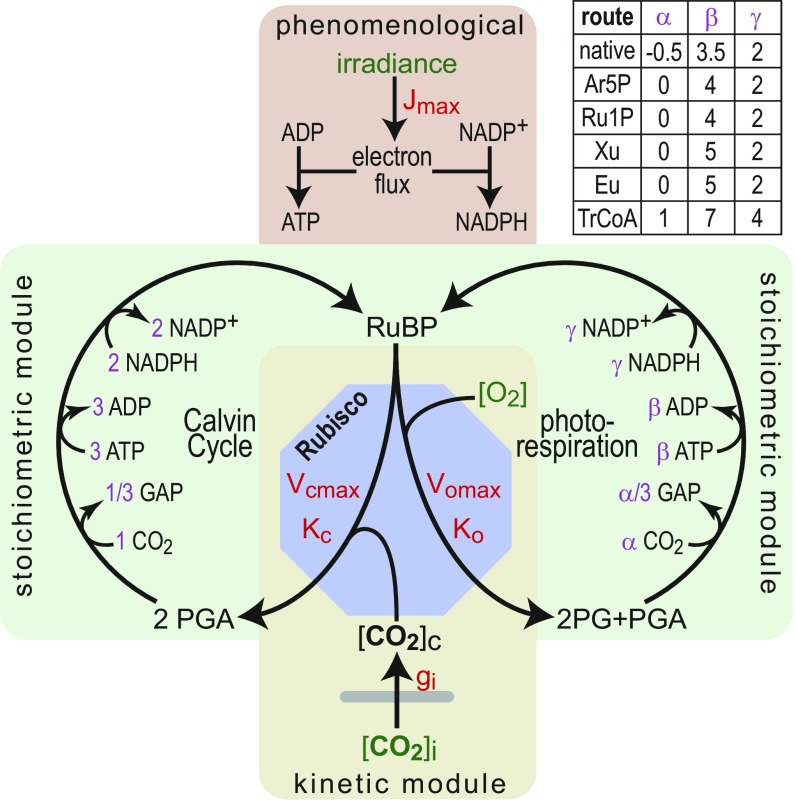
A schematic representation of the kinetic–stoichiometric model. ATP and NADPH biosynthesis is captured by phenomenological equations. CO_2_ diffusion and the activity of Rubisco are modeled kinetically. Consumption of CO_2_ is stoichiometrically linked to the production of GAP, which was used as the product of carbon fixation. The Calvin Cycle and photorespiration are modeled stoichiometrically, accounting for the numbers of CO_2_, ATP, and NADPH that they consume. (*Inset*) The table presents the stoichiometric parameters of native photorespiration as well as the synthetic shunts. Environmental constraints are shown in green, kinetic parameters are shown in red, and stoichiometric parameters are shown in purple. The model is described in detail in *SI Appendix*.

We have extended the classic kinetic–stoichiometric model of leaf photosynthesis in C3 plants (30) to integrate the synthetic shunts and to account for limitation of carbon fixation by enzymes other than Rubisco. Fig. 2 schematically depicts the model, where environmental constraints are indicated in green, kinetic parameters are in red, and stoichiometric parameters are in purple. A full description of the model with all constraints and parameters is given in *SI Appendix*. Our model accounts for the light-dependent rate of ATP and NADPH synthesis (30). The kinetics of CO_2_ diffusion into the stroma as well as Rubisco’s carboxylation and oxygenation reactions are fully modeled using the appropriate kinetic constants and environmental constraints (i.e., O_2_ concentration and intercellular CO_2_ concentration: [CO_2_]_i_ or C_i_). The Calvin Cycle and photorespiration are modeled stoichiometrically, accounting for their consumption of ATP, NADPH, and CO_2_ during the regeneration of RuBP. While the stoichiometry of the Calvin Cycle is fixed—three ATP and two NADPH molecules are consumed to fix one CO_2_ molecule into glyceraldehyde 3-phosphate (GAP)—the stoichiometry of photorespiration changes according to the pathway (table in Fig. 2, *Inset*). As an example, in the following analyses, we use the arabinose 5-phosphate (Ar5P) shunt to represent the carbon-neutral photorespiration bypasses; the other carbon-neutral shunts show very similar results as shown in *SI Appendix*.

In our photosynthesis model, we assume that the carbon fixation rate, *A*, is limited by one of five factors: the rate of ATP supply (*A*_*ATP*_), the rate of NADPH supply (*A*_*NADPH*_), the rate of Rubisco (*A*_*Rubisco*_), the rate of a Calvin Cycle enzyme (*A*_*CBB*_), or the rate of a photorespiration enzyme (*A*_*PR*_). Therefore, we can approximate the carbon fixation rate to

$$A=\min\left(A_{ATP},\;A_{NADPH},\;A_{Rubisco},\;A_{CBB},\;A_{PR}\right).$$ [1]

**Fig. 3.**
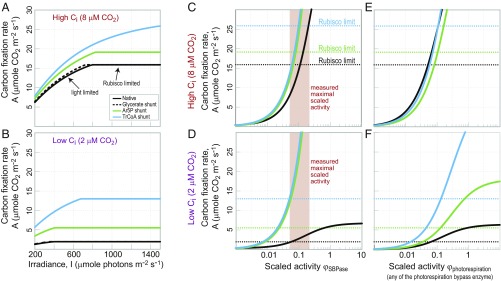
Estimated carbon fixation rate supported by native photorespiration and synthetic bypass. (*A* and *B*) The TrCoA and the Ar5P shunt are expected to support higher carbon fixation rates at all light intensities and intercellular CO_2_ (C_i_) levels. In this analysis, only light and the activity of Rubisco were assumed to limit carbon fixation. The carbon fixation rate supported by a photorespiration bypass that uses the glycerate pathway (12) is shown for comparison. (*C*–*F*) Limitation of carbon fixation rate by Calvin Cycle or photorespiration enzymes at high and low C_i_. The scaled activity, φ, of an enzyme is defined as the activity of the enzyme divided by the maximal carboxylase activity of Rubisco. Dashed lines correspond to the maximal carbon fixation rate as determined by the activity of Rubisco. (*C* and *D*) Limitation of carbon fixation by SBPase. Orange area corresponds to the experimentally measured maximal scaled activity. (*E* and *F*) Limitation of carbon fixation by enzymes of the photorespiration shunt (non-carboxylase enzymes).

We first assume, as in the classic model of Farquhar et al. (30), that only light or Rubisco can be limiting [i.e., carbon fixation rate is limited by ATP availability, NADPH availability, or Rubisco activity (leaving *A*_*CBB*_ and *A*_*PR*_ aside in the initial analysis)]. Fig. 3 shows the light-response curve that our model predicts at high intercellular CO_2_ concentration (Fig. 3*A*) (C_i_ = 8 µM; e.g., stomata are fully open, and CO_2_ can freely enter the intercellular space) and at low intercellular CO_2_ concentration (Fig. 3*B*) (C_i_ = 2 µM; e.g., the stomata are closed as in drought conditions) (31). The light-response curve of carbon fixation using native photorespiration (black line in Fig. 3*A*) corresponds directly to the classical model (30), which is well supported by experimental measurements (32): carbon fixation is light limited up to ∼800 µmol photons m^−2^ s^−1^ and Rubisco limited (light saturated) at higher irradiance. For comparison, we also show the expected curve for the previously suggested photorespiration bypass that uses the glycerate shunt (12), which does not differ much from the native pathway. In contrast, the Ar5P shunt and the TrCoA shunt are expected to support considerably higher carbon fixation rates at all light intensities and intercellular CO_2_ concentrations. Under high CO_2_ conditions, maximal carbon fixation rate is expected to increase by ∼20 or ∼60% when native photorespiration is replaced by the Ar5P shunt or the TrCoA shunt, respectively. Interestingly, when coupled to the TrCoA shunt, carbon fixation is not expected to become light saturated, even at irradiance of up to 1,500 µmol photons m^−2^ s^−1^. Under low CO_2_ conditions, the synthetic pathways are especially advantageous: with the native photorespiration, carbon fixation under low CO_2_ is negligible, while the synthetic shunts could support appreciable carbon fixation rates.

### Required Activity of the Enzymes of the Synthetic Photorespiration Shunts.

Next, we used our model to estimate how the activity of enzymes other than Rubisco—either the Calvin Cycle or photorespiration enzymes—can limit carbon fixation rate. The model and its results are shown in *SI Appendix*. Fig. 3 *C*–*F* presents the main findings. In this analysis, we refer to the scaled activity, φ, of an enzyme defined as the activity of the enzyme (e.g., in micromoles per second per leaf-meter squared) divided by the maximal carboxylase activity of Rubisco (i.e., 80 μmol/s per leaf-meter^2^) (32), which serves as a reference point. Fig. 3 *C* and *D* shows the effect of the scaled activity of sedoheptulose-1,7-bisphosphatase (SBPase) on the carbon fixation rate under high or low C_i_ conditions. Dashed horizontal lines in Fig. 3 *C* and *D* correspond to the expected carbon fixation rates when Rubisco is limiting. For φ values in which the solid lines in Fig. 3 *C* and *D* are lower than the corresponding dashed lines, carbon fixation will be limited by SBPase rather than Rubisco (assuming light-saturating conditions). The red area in Fig. 3 *C* and *D* represents the empirically measured scaled activity of SBPase (33, 34) or, more accurately, its maximal scaled activity, as the measurements were performed (in vitro) with saturating amounts of substrate and a negligible amount of product. This analysis thus captures the well-established limitation of carbon fixation by the activity of SBPase (35): the measured maximal activity of SBPase (33, 34) (in scaled terms: 0.05 < φ_SBPase_ < 0.22) coincides with the shift from SBPase to Rubisco limitation (black solid line intersecting black dashed line within the red area in Fig. 3 *C* and *D*).

As shown by the blue and green curves in Fig. 3 *C* and *D* (which are higher than the black line), the replacement of native photorespiration with the synthetic shunts relieves the limiting effects of SBPase: by using these shunts, the empirically measured activity of SBPase can support higher carbon fixation rate and thus, is less likely to limit photosynthesis. Overall, the synthetic shunts are expected to be less constrained by the activity of normally limiting Calvin Cycle enzymes, such as SBPase. In *SI Appendix*, we extend this analysis to account for all enzymes of the Calvin Cycle.

Next, we turn our attention to the possible limitation of carbon fixation rate by enzymes participating in photorespiration. As shown in Fig. 3*E*, at high C_i_, all photorespiration pathways, native and synthetic alike, present a similar dependency of the carbon fixation rate on φ, such that an enzyme with φ > 0.1 is not expected to limit carbon fixation more than Rubisco does. However, at low C_i_, the native and synthetic pathways diverge (Fig. 3*F*): compared with an enzyme participating in natural photorespiration, an enzyme within the Ar5P shunt or the TrCoA shunt could support a much higher carbon fixation rate for a given φ value. Still, for the enzymes of all routes, φ = 0.1 represents the minimal value required such that they would not limit carbon fixation (more than Rubisco does).

With respect to the TrCoA shunt, the activity of glycolyl-CoA carboxylase depends on the availability of inorganic carbon. Therefore, the φ-dependent analysis does not accurately capture the required enzyme activity. In *SI Appendix*, we present a dedicated analysis of this enzyme and show that its scaled activity needs to be threefold higher than that of Rubisco to not limit carbon fixation. Also in *SI Appendix*, we present a full kinetic model for the synthetic bypasses, taking into account all relevant parameters, including maximal rates, affinities toward reactants, thermodynamics, and physiological concentrations of metabolites and cofactors (36). This analysis shows that engineering enzymes for a catalytic rate high enough to not limit carbon fixation rate is a feasible task given the characteristic kinetic parameters of corresponding enzyme families.

### Design Goals for Engineering the Glycolate Reduction Module.

Next, we aimed to experimentally realize a carbon-neutral shunt and to this end, engineer the enzymatic activities needed for its key element: the glycolate reduction module. Since glycolate is similar to acetate and propionate, the substrates of known CoA-ester synthetases and reductases, enzyme engineering could be readily applied to obtain the required catalytic efficiency with glycolate. However, in addition to catalytic efficiency, there are two important design constraints for the reduction module. First, glycolyl-CoA reduction is reversible as are all known CoA-ester reductions (37), and in most cells, concentrations of NAD^+^ are much higher than NADH, sometimes by a few orders of magnitude (38). Thus, an NADH-dependent reductase would promote the oxidation of glycolaldehyde to yield glycolyl-CoA. Since NADPH is directly produced by photosynthesis and also serves as the redox source for carbon fixation and photorespiration, an NADPH-driven reduction would enable a direct transfer of photosynthetic reducing power toward the engineered photorespiration bypass. Thus, to realize a carbon-neutral photorespiration bypass, the reductase should be engineered to preferentially use NADPH over NADH so that reduction is favored over oxidation. Second, the reductase should have reduced activity toward acetyl-CoA to avoid reduction of this central metabolic intermediate to yield acetaldehyde, which may react nonspecifically with other cellular components. Such selectivity, however, is challenging from an enzyme engineering perspective: discrimination against a larger substrate is often readily achieved by introducing active site mutations that sterically block the undesired larger substrate, but discrimination against a smaller substrate (i.e., exclusion of the acetyl moiety in an active site shaped for the larger glycolyl moiety) is far from trivial (39).

### Engineering a Glycolyl-CoA Synthetase.

*Escherichia coli* acetyl-CoA synthetase (*Ec*ACS) ligates acetate to CoA while hydrolyzing ATP to AMP (40), and a nearly identical ACS was reported to have promiscuous activity with propionate (41), suggesting that *Ec*ACS may also accept glycolate. We tested *Ec*ACS and observed ligation of glycolate to CoA, but the catalytic efficiency was much reduced relative to acetate (∼4,000-fold lower *k*_cat_/*K*_M_) (Table 1). ACS, therefore, required engineering for higher glycolyl-CoA and lower ACS efficiency. One potential problem in engineering natural enzymes is that they are only marginally stable, thus limiting the accumulation of function-altering mutations (42). Starting from the molecular structure of the homologous *Salmonella enterica* acetyl-CoA synthetase (*Se*ACS) (43), we applied “Protein Repair One-Stop Shop” (PROSS), a protein stability design algorithm that uses sequence and structural information to predict stable protein variants (44), and tested two designs (45). One design, dubbed ACSstab (ACS_PROSS in ref. 45), encoded 48 mutations relative to *Se*ACS and 57 relative to *Ec*ACS, and it exhibited similar catalytic efficiency to *Ec*ACS with both glycolate and acetate (Table 1). ACSstab was also significantly more stable: whereas *Ec*ACS was inactive after a 10-min incubation at >50 °C, ACSstab retained full activity (*SI Appendix*, Fig. S1).

**Table 1.** Kinetic parameters of acyl-CoA synthetase variants

Kinetic parameters	Substrate	*Ec*ACS	ACSstab	ACS19
*k*_cat_/*K*_M,_ s^−1^ M^−1^	Glycolate	41.2 ± 0.3	38.0 ± 1.4	82.1 ± 1.7
*k*_cat_, s^−1^	Acetate	13.5 ± 0.1	33.9 ± 0.5	19.6 ± 0.4
*K*_M_, µM		87 ± 4	160 ± 4	980 ± 40
*k*_cat_/*K*_M_, s^−1^ M^−1^		1.55 ± 0.05 × 10^5^	2.12 ± 0.03 × 10^5^	2.00 ± 0.05 × 10^4^

*Ec*ACS is Gene ID 948572. ACSstab is a variant of *Se*ACS engineered for higher stability with no active site alterations. Engineered ACS19 is a variant of ACSstab with five active site mutations (V310I, S314A, Y355F, V386T, and F421C). Rates were measured via AMP production using a coupled assay with myokinase, pyruvate kinase, and lactate dehydrogenase. Reactions were carried out at saturating CoA (1 mM) in 50 mM Hepes, pH 8.0, 2.5 mM ATP, 5 mM MgCl_2_, 1 mM DTT, 0.6 mM NADH, and 2.5 mM phospholenolpyruvate at 37 °C. Average of at least three independent experiments; ± indicates SE. For all ACS variants, *K*_M_ on glycolate was in excess of 50 mM, and therefore, only *k*_cat_/*K*_M_ derived from the Michaelis–Menten curve fit is shown. *SI Appendix* has additional details.

**Fig. 4.**
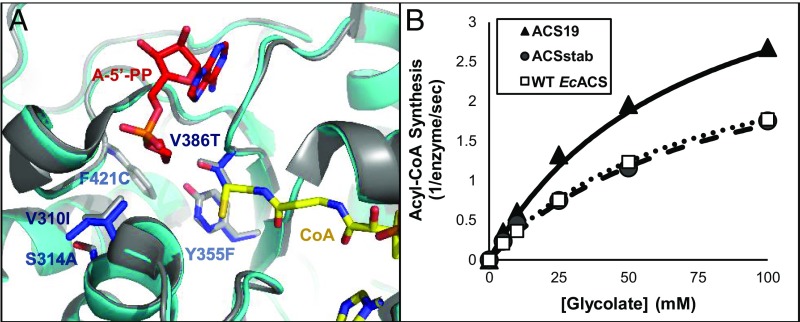
The engineered glycolyl-CoA synthetase. (*A*) The computationally designed variant ACS19 (blue) has five mutations in and around the carboxylic acid binding pocket. The model based on PDB ID code 1PG4 is shown in gray (22). CoA is shown in yellow, and competitive inhibitor adenosine-5′-propylphosphate (A-5′-PP) is shown in red. (*B*) ACS19 exhibits improved glycolyl-CoA synthesis. Kinetics were monitored by ATP consumption with a coupled assay of myokinase, pyruvate kinase, and lactate dehydrogenase. Reactions were carried out with 1 mM CoA, 2.5 mM ATP, and 0.1 μM ACS. Activity is reported as reaction turnover per second per enzyme monomer. Shown here and in other figures is the average of two or more independent measurements, and error bars show SEM (too small to be seen here). WT, wild type.

We recently described a method, called FuncLib, for computational design of small yet effective enzyme repertoires with diverse active sites (45). Applied to ACSstab, FuncLib provided variants with large changes in substrate selectivity, foremost with improved efficiency relative to large substrates, such as butyrate. We tested whether the FuncLib designs also showed improved activity relative to glycolate and found that design ACS19 exhibited modestly improved kinetic parameters for glycolate (Table 1). ACS19 had two mutations in the substrate binding pocket, V310I and V386T, and three adjacent mutations, S314A, Y355F, and F421C (Fig. 4*A*). Compared with *Ec*ACS, ACS19 had approximately 2-fold improved *k*_cat_/*K*_M_ for glycolate (Fig. 4*B*) and 8-fold lower *k*_cat_/*K*_M_ for acetate (Table 1), translating to a 16-fold shift in favor of glycolate, although acetate remained by far the preferred substrate (∼250-fold higher *k*_cat_/*K*_M_).

### Engineering a Glycolyl-CoA Reductase.

*Rhodopseudomonas palustris* propanediol utilization protein (*Rp*PduP) reduces propionyl-CoA to propionaldehyde and shows promiscuous activity with acetyl-CoA and other CoA-ester substrates (46). A molecular structure of *Rp*PduP was available, enabling structure-based design. We expressed and purified *Rp*PduP in *E. coli* and compared its activity with glycolyl-CoA and acetyl-CoA as well as its selectivity for the native cofactor NADH over NADPH (Table 2). *Rp*PduP exhibited activity with glycolyl-CoA and NADPH but at an ∼120-fold lower catalytic efficiency in comparison with acetyl-CoA and NADH. Nevertheless, the observed promiscuous activity suggested that *Rp*PduP could be engineered into an NADPH-dependent glycolyl-CoA reductase (GCR).

**Table 2.** Kinetic parameters of acyl-CoA reductase variants

Kinetic parameters	Substrate	*Rp*PduP	Engineered GCR
*k*_cat_, s^−1^	Glycolyl-CoA	4.8 ± 0.4 (NADH)	1.6 ± 0.2 (NADH)
		0.40 ± 0.06 (NADPH)	2.7 ± 0.3 (NADPH)
K_M_, mM		0.22 ± 0.12 (NADH)	0.24 ± 0.02 (NADPH)
*k*_cat_, s^−1^	Acetyl-CoA	15.3 ± 0.4 (NADH)	0.63 ± 0.03 (NADH)
		2.7 ± 0.2 (NADPH)	1.4 ± 0.1 (NADPH)
*K*_M_, mM		0.22 ± 0.02 (NADPH)	0.22 ± 0.06 (NADPH)
*k*_cat_, s^−1^	NADH	15.3 ± 0.4 (acetyl-CoA)	0.63 ± 0.03 (acetyl-CoA)
		4.8 ± 0.4 (glycolyl-CoA)	1.6 ± 0.2 (glycolyl-CoA)
*K*_M_, mM		0.12 ± 0.03 (acetyl-CoA)	0.07 ± 0.01 (acetyl-CoA)
*k*_cat_, s^−1^	NADPH	2.7 ± 0.2 (acetyl-CoA)	1.4 ± 0.1 (acetyl-CoA)
		0.40 ± 0.06 (glycolyl-CoA)	2.7 ± 0.3 (glycolyl-CoA)
*K*_M_, mM		0.37 ± 0.09 (glycolyl-CoA)	0.24 ± 0.06 (glycolyl-CoA)

GCR is a variant of PduP containing eight active site mutations (P224G, I259R, N263L, I282L, L328I, P329T, V331T, and L483H). The second substrate [either acyl-CoA or NAD(P)H] was used in saturation and is written in parentheses. Reactions were carried out in 50 mM Hepes, pH 8.0, 5 mM ATP, and 5 mM MgCl_2_ at 37 °C. Activity was measured by the rate of NAD(P)H oxidation using change in absorbance at 340 nm. Average of at least three independent experiments, ± indicates SE (*SI Appendix*).

**Fig. 5.**
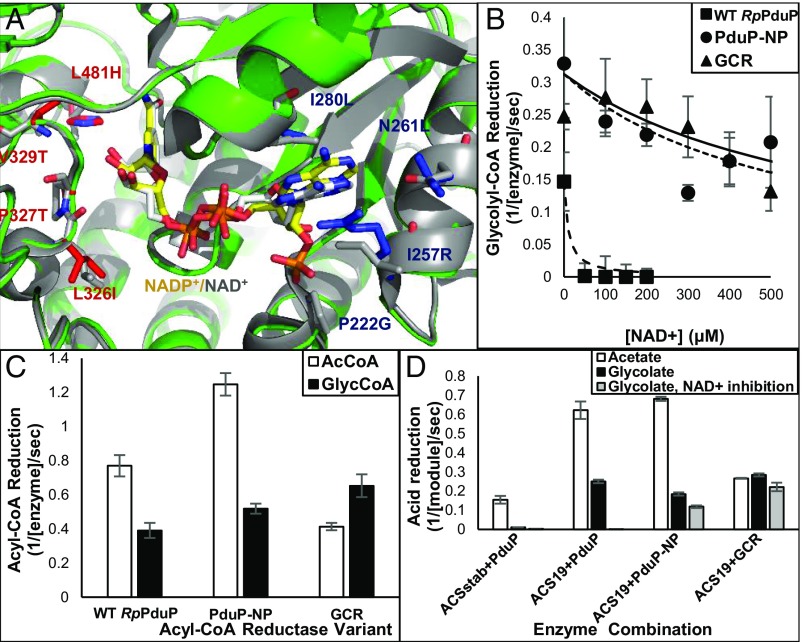
Engineering of *Rp*PduP for improved NADPH-dependent glycolyl-CoA reduction. (*A*) The active site of the engineered GCR (PDB ID code 6GVS; green) compared with wild-type (WT) *Rp*PduP [PDB ID code 5JFL; gray (65)]. Sites targeted for NADPH selectivity are shown in blue, and sites targeted for glycolyl-CoA selectivity are shown in red; NADP^+^ bound to GCR is shown in yellow, and NAD^+^ bound to *Rp*PduP is shown in gray. (*B*) NADPH preference of the evolved GCR variants (PduP-NP and GCR) permits reduction despite high NAD^+^ levels (0.4 μM enzyme, 250 μM glycolyl-CoA, 500 μM NADPH; rates were derived from change in absorbance at 340 nm, and activity is reported as reaction turnovers per second per enzyme monomer). (*C*) The evolved GCR preferentially reduces glycolyl-CoA over acetyl-CoA (0.4 μM enzyme, 500 μM acyl-CoA, 500 μM NAPDH). (*D*) The two-enzyme module efficiently reduces glycolate to glycolaldehyde in the presence of NAD^+^. WT and engineered enzymes (0.2 μM each) were incubated with 20 mM acetate or glycolate, 250 μM CoA, and 1 mM NADPH with or without NAD^+^ (1 mM). The reduction rate was maximal within first 20 min and is reported as turnovers per second per enzyme pair.

We first targeted the NADH binding pocket at the site where the cofactor’s 2′-phosphate group typically resides in NADPH-dependent reductases. We used a combination of structure-based design and the library design for NADP/NAD switches proposed by Cahn et al. (47). We constructed a combinatorial library with mutations at positions P222, I257, and T260 of *Rp*PduP followed by single-site saturation mutagenesis libraries around the entire binding pocket (Fig. 5*A*). The mutant libraries were expressed in *E. coli*, and crude-lysate screening was carried out in 96-well plates using acetyl-CoA and either NADPH or NADH (at this stage, glycolyl-CoA activity was too low for detection). The variant with the highest specificity toward NADPH had four mutations: P222G, I257R, N261L, and I280L. Foremost, this NADPH-preferring PduP variant, PduP-NP, was able to drive the NADPH-mediated reduction reaction in the presence of equimolar NAD^+^ competing as oxidizing cofactor (Fig. 5*B*). In contrast, the reduction reaction by the wild-type *Rp*PduP was completely inhibited in the presence of NAD^+^ at even 1/10th of the concentration of NADPH, favoring the competing oxidation reaction. Moreover, the PduP-NP variant was still able to use glycolyl-CoA as a substrate, thus providing a basis for additional improvements. However, PduP-NP, like the wild-type *Rp*PduP, also exhibited a higher activity with acetyl-CoA (Fig. 5*C*), an undesirable activity that produces acetaldehyde.

To improve selectivity toward glycolyl-CoA, we diversified the L326, P327, and V329 sites that form the first shell of *Rp*PduP’s acyl binding site, introducing every amino acid present in orthologous PduP sequences (48). We assayed for both glycolyl-CoA and acetyl-CoA reduction activity and selected variants that had high activity on glycolyl-CoA and decreased activity on acetyl-CoA. We then carried out a second round of saturation mutagenesis at second-shell sites (T196, N197, A296, L481) and identified a variant with four mutations in the acyl binding pocket (L326I, P327T, V329T, and L481H) in addition to the four mutations in the NADPH site (Fig. 5*A*). This variant, named GCR exhibited NADPH preference as well as ∼12-fold improved selectivity for glycolyl-CoA over acetyl-CoA compared with wild-type *Rp*PduP (in terms of *k*_cat_/*K*_M_ ratios with NADPH) (Table 2). GCR could function in the presence of equimolar NAD^+^ (Fig. 5*B*) and had reduced activity on acetyl-CoA at the expected submillimolar cellular concentrations (Fig. 5*C*).

To investigate the specificity changes in GCR, we obtained an X-ray crystal structure of GCR with bound NADP^+^ (Fig. 5*A*) [Protein Data Bank (PDB) ID code 6GVS]. We found that GCR binds NADP^+^ in a similar manner to how *Rp*PduP binds NAD^+^ (PDB ID code 5JFL). In GCR, R257 (Ile in wild-type *Rp*PduP) facilitates NADP(H) binding by a π-stacking interaction with the adenine ring and also, a charge–charge interaction with the 2′-phosphate group (*SI Appendix*, Figs. S4 and S5*A*). However, the GCR crystal structure also revealed alternative binding modes where the adenosine-2′-phosphate moiety assumes different positions (*SI Appendix*, Figs. S4 and S5*B*). Nevertheless, in these cases, the nicotinamide moiety is still correctly positioned to catalyze the reduction reaction (*SI Appendix*, Fig. S4), and this structural heterogeneity may contribute to GCR’s cofactor promiscuity. Modeling glycolyl-CoA binding based on an alignment with propionyl-CoA–bound *Rp*PduP (PDB ID code 5JFM) suggests that the P327T, L481H, and V329T mutations facilitate binding by forming a hydrogen bond network with the hydroxyl group of glycolyl-CoA (*SI Appendix*, Fig. S6).

### The Combined Glycolate Reduction Module.

We next examined whether the combination of the two engineered enzymes could efficiently convert glycolate to glycolaldehyde. We compared the activity of wild type-like ACSstab and wild-type *Rp*PduP with that of engineered ACS19 in combination with different engineered PduP variants (Fig. 5*D*). In the presence of NADPH as the sole reducing cofactor, the wild-type combination showed no detectable reduction of glycolate. When ACS19 was combined with *Rp*PduP, glycolate reduction was observed but was suppressed on addition of NAD^+^. Replacing wild-type *Rp*PduP with the NADPH-preferring PduP-NP restored activity in the presence of NAD^+^, but reduction of acetate was almost four times faster than reduction of glycolate. When ACS19 was combined with GCR, glycolate was efficiently reduced, reduction of acetate was diminished, and glycolate reduction was barely inhibited by NAD^+^ (Fig. 5*D*). None of these occurred with the wild-type pair. Engineered ACS19 and GCR, therefore, comprised the glycolate reduction module.

### Glycolate Metabolism to RuBP.

Of the three alternative carbon-neutral bypasses, the aldol condensation of glycolaldehyde with GAP to form Ar5P represents the simplest way for glycolaldehyde assimilation to the Calvin Cycle. The *E. coli* fructose 6-phosphate aldolases FsaA and FsaB were reported to use glycolaldehyde as a preferred nucleophilic donor and GAP as a preferred acceptor, thus yielding Ar5P (25, 49). The subsequent isomerization to ribulose-5-phosphate could be catalyzed by *E. coli* isomerase KdsD or GutQ (50, 51). Phosphorylation of ribulose-5-phosphate to form RuBP is part of the canonical Calvin Cycle, and *Rhodobacter sphaeroides* phosphoribulokinase (*Rs*PRK) (52) was used here.

**Fig. 6.**
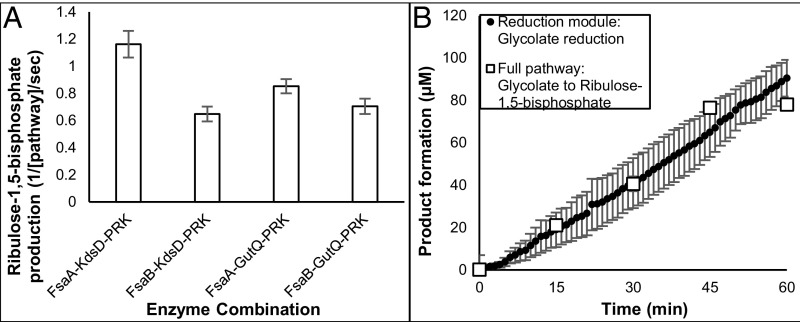
In vitro realization of a carbon-conserving photorespiration pathway. (*A*) The glycolaldehyde assimilation module. Aldolases (*Ec*FsaA or *Ec*FsaB), isomerases (*Ec*KdsD or *Ec*GutQ), and ribulose 5-phosphate kinase (*Rs*PRK) were combined, and RuBP production from glycolaldehyde and glyceraldehyde-3-phosphate was monitored by liquid chromatography–mass spectrometry (0.5 μM each enzyme, 2 mM glycolaldehyde, 5 mM ATP, 1 mM glyceraldehyde-3-phosphate; shown are turnovers per second per pathway in the first 10 min). (*B*) The complete Ar5P pathway. The glycolate reduction module and glycolaldehyde assimilation modules were combined. Absorbance at 340 nm reported the reduction rate and by liquid chromatography–mass spectrometry, was used to measure RuBP formation (0.5 μM of each enzyme except for *Rs*PRK, which was 2 μM; 10 mM glycolate; 1 mM NADPH; 5 mM ATP; 250 μM CoA; 1 mM glyceraldehyde-3-phosphate).

We tested different combinations of the above enzymes: in all cases, glycolaldehyde and GAP were consumed to produce RuBP at detectable amounts. *Ec*FsaA, *Ec*KdsD, and *Rs*PRK exhibited the highest yield at an average initial rate of ∼1.2 turnovers per second per enzyme for the entire glycolaldehyde assimilation module (Fig. 6*A*). Omitting any of these three enzymes abolished RuBP production. Finally, this module (*Ec*FsaA-*Ec*KdsD-*Rs*PRK) was combined with the engineered glycolate reduction module to test whether glycolate could be converted to RuBP. RuBP was produced from glycolate with an overall turnover rate of 0.05/s per enzyme for the entire pathway. The rate of glycolate reduction as measured by NADPH consumption matched the rate of RuBP production, indicating tight coupling between the reduction and condensation modules, with no waste of reducing potential or off-target reactions in this in vitro setup (Fig. 6*B*).

## Discussion

State-of-the-art metabolic engineering is mostly based on grafting a natural pathway into a different organism: for example, in the biosynthesis of plant natural products (53). Other studies “mix-and-match” enzymes from different pathways that act on their native substrates (54). While these approaches can tackle important challenges, new reactions and substrates can significantly expand the potential space of metabolic pathways (55). Using this strategy, we put forward five alternatives to natural photorespiration, four of which involve glycolate reduction to glycolaldehyde that is assimilated into the Calvin Cycle without carbon loss (Fig. 1 *A*–*D*), while one pathway, the TrCoA shunt (Fig. 1*E*), directly assists carbon fixation. Our computational model indicates that these synthetic bypasses could boost carbon fixation rate at all relevant light intensities and CO_2_ levels (Fig. 3).

The glycolaldehyde assimilating pathways (Fig. 1 *A*–*D*) are, in principle, more amenable to implementation than the carbon-fixing TrCoA shunt (Fig. 1*E*). Among these pathways, the main advantage of the Ar5P shunt (Fig. 1*A*) is the very high affinity and rate for glycolaldehyde condensation by fructose 6-phosphate aldolase: *K*_M_ = 0.2 mM and *k*_cat_ = 16 s^−1^ (56). That said, the Ar5P shunt lacks an irreversible reaction immediately downstream of glycolaldehyde. Therefore, glycolaldehyde concentration is expected to be in a steady state with the intermediates of the Calvin Cycle. Considering the measured concentration of xylulose 5-phosphate, the Ar5P shunt could probably operate only at [glycolaldehyde] >1 mM (*SI Appendix*). The erythrulose shunt and xylulose shunt harbor irreversible reactions and are, therefore, expected to efficiently operate even if [glycolaldehyde] <1 mM. The erythrulose pathway has a major advantage over the xylulose pathway: transketolase accepts glycolaldehyde at a higher rate and affinity than transaldolase. The former enzyme is characterized by *V*_max_ = 60 μmol/min per milligram and *K*_M_ = 13 mM (26), while the latter enzyme exhibits 1 < *V*_max_ < 9 μmol/min per milligram and 70 < *K*_M_ < 100 mM (27). We note that l-erythrulose assimilation to erythrose 4-phosphate is well documented and that the enzymes involved were shown to have high activity (55). The main advantage of the Ru1P shunt is the recycling of 2PG directly and irreversibly into RuBP, thus bypassing the entire Calvin Cycle. The realization of this synthetic pathway awaits the engineering of another enzyme—Ru1P 5-kinase. However, so far, this activity was undetectable in >20 sugar and phosphosugar kinases that we have tested.

While none of the glycolaldehyde assimilation routes are obviously superior, they all rely on glycolate reduction to glycolaldehyde. The engineering of the glycolate reduction module had to meet specific challenges that are not necessarily encountered in a nonmetabolic context: the engineered enzymes had to show sufficiently high rates, a specific cofactor preference, and selectivity to suppress undesired reactions. The primary requirement for a glycolyl-CoA synthetase is high turnover rate. Glycolate rates of production in photorespiratory conditions are very high (57), while acetate is generally present at submillimolar concentrations (58); therefore, high substrate selectivity is not required in this case. We explored a repertoire of previously designed ACS variants and found that ACS19 has improved catalytic efficiency with glycolate and higher stability.

The engineering of a GCR was a more complex task. Cofactor balance is essential for pathways involving redox reactions (59). Engineering enzymes to change NAD(P) preference has been demonstrated in quite a few cases, and some engineering rules have been described (47). However, NADH to NADPH switches are comparatively rare. Most studies began with enzymes exhibiting relatively moderate NADPH/NADH cofactor preferences (generally ranging from 0.1 to 10). Wild-type *Rp*PduP had a 16-fold preference for NADH over NADPH. Our final engineered variant, GCR, exhibited a 1.1-fold preference for NADPH over NADH (Fig. 5*A* and Table 2). Although the evolved enzyme accepts both NADH and NADPH, the obtained shift in specificity was sufficient to prevent the reversal of the reaction even in the presence of equimolar NAD^+^ (Fig. 5 *B* and *D*). We were also successful in increasing the catalytic efficiency for glycolyl-CoA reduction by approximately sevenfold and shifting the substrate selectivity of *Rp*PduP from preferring acetyl-CoA over glycolyl-CoA (sixfold) to a twofold preference for glycolyl-CoA.

In general, engineering enzymes to discriminate against smaller substrates is challenging and often results in significantly lower rates (39). Nonetheless, it was possible to engineer GCR to have both increased rate and higher selectivity toward NADPH and glycolyl-CoA, although most variants seen in our screens had either improved rate and decreased selectivity or the reverse. The X-ray crystal structure of GCR shows that binding to NADPH is facilitated by interaction of R257 with the 2′-phosphate of NADPH (*SI Appendix*, Figs. S4 and S5) and that binding to glycolyl-CoA is mediated by three mutations to polar amino acids (T327, T329, and H481), which may form two new hydrogen bonds with the hydroxyl group of the glycolyl moiety (*SI Appendix*, Fig. S6). The structure of GCR may facilitate additional engineering of acyl-CoA reductases to better utilize NADPH (60), both for synthetic photorespiration and for other nonnatural assimilation pathways (61).

Our computational model predicts that enzyme activity of about 1/10th that of Rubisco (carboxylation rate) would suffice to support the required photorespiration flux. The *k*_cat_ of GCR with glycolyl-CoA and NADPH is similar to that of Rubisco (∼3 s^−1^), and its affinity toward these substrates is not limiting (*K*_M_ < 0.5 mM). Hence, expressing GCR at 1/10th of the expression level of Rubisco is expected to sustain sufficient activity. However, the biosynthesis of glycolyl-CoA at the physiologically relevant glycolate concentration of 10 mM proceeds only at ∼0.7 s^−1^; therefore, if 1/10th of Rubisco expression level is the desirable target, the activity of the glycolyl-CoA synthetase would have to be increased approximately fivefold.

How would carbon-conserving photorespiration pathways affect plant growth and agricultural productivity? While the answer strongly depends on the crop in question and the environmental conditions, CO_2_ enrichment experiments suggest that enhanced carbon fixation can increase growth and above-ground production (62). Similarly, the introduction of inorganic carbon transporters, intended to increase CO_2_ availability for Rubisco, led to enhanced growth and grain yield (63). Furthermore, overexpression of enzymes that limit carbon fixation (e.g., SBPase) was shown to increase biomass by up to 30% (35). Theoretically, the synthetic pathways described here are expected to support a 10–60% increase in carbon fixation rate, which could provide a dramatic boost in plant growth and productivity. The in vitro realization of a carbon-conserving pathway, as provided in this study, provides the foundation for eventual realization in photosynthetic organisms.

## Materials and Methods

Described here are the key elements; additional details are provided in *SI Appendix*.

### pathSeekR Algorithm: Searching for Photorespiration Pathways.

The pathSeekR algorithm was developed to search for pathways in a reaction network, starting with a source compound and ending in a set of sink compounds. In the context of photorespiration shunts, the source is 2PG, and the sink comprises all Calvin Cycle intermediates. pathSeekR creates a reaction network where the nodes represent the compounds and the (directed) edges correspond to the reactions (a reversible reaction is represented by two edges). A compound space is created by combining all possible chemical groups up to a maximal number of groups. The space is pruned by removing compounds that contravene biochemical rules (e.g., adjacent carbonyl groups that are highly reactive). The chemical groups of pathSeekR are achiral (i.e., all stereoisomers are represented by a single compound). Cofactors and inorganic substrates are ignored. Each reaction is an implementation of a simple rule that captures a naturally occurring enzymatic mechanism. A rule is defined as a pair of chemical patterns—a sequence of chemical groups: one for the substrate and one for the product. A reaction is implemented by substituting the substrate pattern with the product pattern. pathSeekR searches the network until a certain number of pathways are generated or until a certain depth (number of reactions) is reached. In this study, we restricted the solutions to a maximal depth of nine nodes (including source and sink), which is equivalent to eight reactions. A comprehensive description of the algorithm as well as a complete set of reaction rules are given in *SI Appendix*.

### The Stoichiometric–Kinetic Model.

The classical photosynthesis model of Farquhar et al. (30) was extended to include the stoichiometry of the synthetic photorespiration bypasses and to account for possible limitation of the carbon fixation rate by an enzyme of the Calvin Cycle or photorespiration. The Calvin Cycle as well as the native and synthetic photorespiration routes were treated as isolated cycles that regenerate RuBP and either fix or release a stoichiometric amount of CO_2_ while consuming stoichiometric amounts of ATP and NADPH. Kinetics of Rubisco were explicitly modeled assuming that it is always saturated with respect to RuBP and that the concentration of RuBP exceeds that of Rubisco. The light reactions were modeled phenomenologically. Limitation of carbon fixation rate by an enzyme of the Calvin Cycle or photorespiration was derived as a function of a single-enzyme parameter φ, which corresponds to the enzyme rate expressed in units of the maximal carboxylation rate of Rubisco. In the modeling of the photorespiratory carboxylase, φ was further scaled by enzyme saturation with bicarbonate, which was assumed to be in equilibrium with dissolved CO_2_; affinity of the carboxylase toward bicarbonate was assumed to be 1 mM. A comprehensive description of the model and its results are given in *SI Appendix*.

### Enzyme Cloning and Purification.

*Ec*FsaA, *Ec*FsaB, *Ec*GutQ, *Ec*KdsD, and *Ec*ACS with N-terminal 6xHis tags were obtained from the ASKA collection in the pCA24N vector (64). *Rs*PRK was cloned with a C-terminal 6xHis tag into pET21a. *Rp*PduP was cloned into pET45a with an N-terminal 6xHis tag (46). The engineered ACSstab gene was synthesized by Twist Bioscience and cloned into pET21a with a C-terminal 6xHis tag. Cloning was carried out by restriction ligation. All vectors were transformed into *E. coli* BL21(DE3) by electroporation. Protein expression was induced by isopropyl β-D-1-thiogalactopyranoside, and purification was carried out using Nickel-NTA beads. ACS preparations were treated with *E. coli* deacetylase CobB, which was also expressed and purified from the ASKA collection. Deacetylation conditions were 1 mM NAD^+^, 50 mM Hepes, pH 8, 100 mM NaCl, 2 mM MgCl_2_, 20 μM ACS, and 4 μM CobB at 37 °C overnight. *SI Appendix* has sequences and additional details regarding all methods.

### Synthesis of Glycolyl-CoA.

Glycolyl-CoA was synthesized from glycolate and CoA using carbonyldiimidazole. The reaction mixture was either used directly for library screening or purified using preparative HPLC to ∼90% purity.

### Enzyme Activity Assays.

For acyl-CoA synthetases, a 200 μL reaction mixture containing 50 mM Hepes, pH 8, 5 mM MgCl_2_, 1 mM DTT, 1 mM CoA, 2.5 mM ATP, 0.6 mM NADH, 2.5 mM phospholenolpyruvate, 15 U/mL pyruvate kinase, 23 U/mL lactate dehydrogenase, 25 U/mL myokinase, and various concentrations of carboxylic acids was incubated with enzyme (0.05–0.1 μM) in a UV-transparent 96-well microplate, and absorbance at 340 nm was monitored at 37 °C for 10 min.

For acyl-CoA reductases, 200 μL reaction mixtures containing 50 mM Hepes, pH 8, 5 mM MgCl_2_, 1 mM DTT, and various concentrations of acyl-CoA and NAD(P)H were incubated with enzyme (0.05–0.5 μM) in a UV-transparent 96-well microplate at 37 °C, and NAD(P)H consumption was monitored at 340 nm. For the glycolate reduction module, the components of both assays were combined in 200 μL, and reduction was monitored by NADPH consumption.

### Liquid Chromatography–Mass Spectrometry.

A 100 μL reaction mix was cleared of protein by filtration, and samples were run on a Waters e2695 Separation module with a Phenomenex Luna-NH_2_ column (3 µm, 100 Å) and detected on a Waters Acquity QDA system. RuBP (>99.0% purity) was used as a standard.

### Crystallization and Structure Determination of the Glycolyl-CoA Reductase.

More detailed procedures are in *SI Appendix*. Crystals were soaked briefly with mother liquor supplemented with 30% (vol/vol) ethylene glycol and 10 mM NADP^+^ before freezing in liquid nitrogen. X-ray diffraction data were collected at the beamline ID30B of the European Synchotron Radation Facility (ESRF) and at the beamline P13 of the Deutsches Elektronen-Synchrotron (DESY). The structures were solved by molecular replacement using the structure of the wild-type propionaldehyde dehydrogenase (46) from *R. palustris* (PDB ID code 5JFL) as the search model. The GCR structure model was deposited at the PDB in Europe under PDB ID code 6GVS.

## Supplementary Material

Supplementary File
