# Supplementary material for: Design and in vitro realization of carbon-conserving photorespiration
Source: Proc Natl Acad Sci U S A. 2018 Nov 20;115(49):E11455–64. doi: 10.1073/pnas.1812605115 (PMC6298115; doi:10.1073/pnas.1812605115)
Supplement: Supplementary File [file pnas.1812605115.sapp.pdf]

Supplemental Experimental Results, Materials, and Methods

Design and *in vitro* realization of carbon-conserving photorespiration

Devin L. Trudeau[1‡], Christian Edlich-Muth[2‡], Jan Zarzycki[3], Marieke Scheffen[3], Moshe Goldsmith[1], Olga Khersonsky[1], Ziv Avizemer[1], Sarel J. Fleishman[1], Charles A.R. Cotton[2], Tobias J. Erb[3], Dan S. Tawfik[1*], Arren Bar-Even[2*]

[1]Department of Biomolecular Sciences, Weizmann Institute of Science, 234 Herzl Street, Rehovot 7610001, Israel

[2]Max Planck Institute of Molecular Plant Physiology, Am Mühlenberg 1, 14476 Potsdam-Golm, Germany

[3]Max Planck Institute for Terrestrial Microbiology, Karl-von-Frisch-Straße 10, D-35043 Marburg, Germany

www.pnas.org/cgi/doi/10.1073/pnas.1812605115

Supplemental Experimental Results

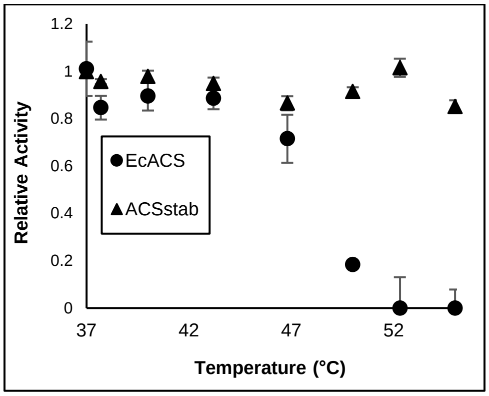

Figure S1. Thermostability of *E. coli* ACS and its PROSS variant ACSstab. *Ec*ACS, *E. coli* acetyl-CoA synthetase (Gene ID: 948572); ACSstab is a variant of *S. enterica* ACS engineered for higher stability with no active-site alterations; DNA and amino acid sequences are provided in **Table S4**. The rate of acetyl-CoA synthesis by *Ec*ACS and ACSstab ware measured after a 10 min incubation at various temperatures. Reactions were carried out with 0.05 μM enzyme, 0.5 mM acetate, 250 μM CoA, 5 mM ATP, and activity was measured by titrating the unreacted CoA using DTNB. Shown are residual activities compared to a sample incubated at ambient temperature (average of three measurements and standard error).

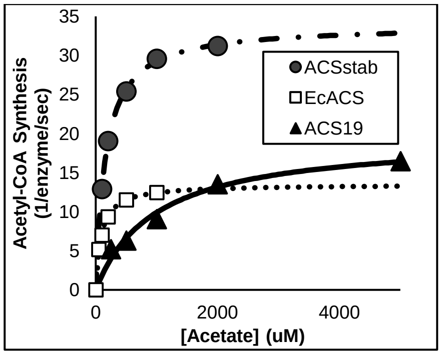

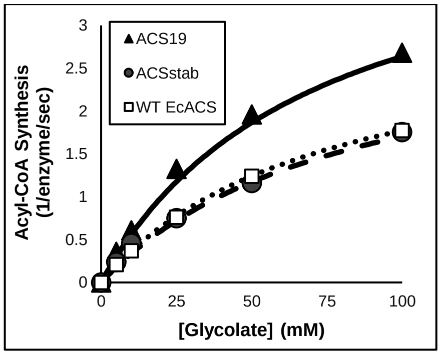

Figure S2. Michaelis-Menten curves for the acyl-CoA synthetase variants. *Ec*ACS, *E. coli* acetyl-CoA synthetase (Gene ID: 948572); ACSstab is a variant of *S. enterica* ACS engineered for higher stability with no active-site alterations. Engineered ACS19 is a variant of ACSstab with five active site mutations (V310I, S314A, Y355F, V386T, and F421C; DNA and amino acid sequences are provided in **Table S4).** Rates were measured by AMP production using a coupled assay with myokinase, pyruvate kinase, and lactate dehydrogenase. Reactions were carried out in 50 mM HEPES pH 8, 2.5 mM ATP, 5 mM $MgCl_2$, 1 mM DTT, 1 mM CoA, 0.6 mM NADH, 2.5 mM phospholenolpyruvate at 37 °C. ACS concentration was 0.05-0.1 μM. Average of at least three independent experiments; ± indicates standard error.

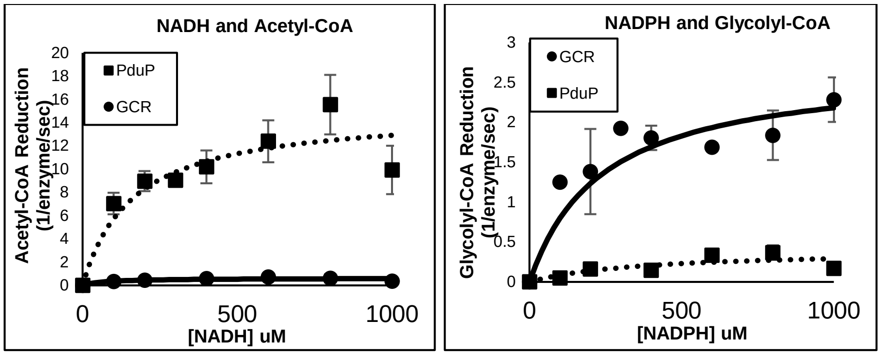

Figure S3. Michaelis-Menten curves for PduP and GCR with either NADH or NADPH as cosubstrate. PduP is *Rhodopseudomonas palustris* propionyl-CoA reductase, GCR is a variant of PduP with eight active site mutations: P222G, I257R, N261L, I280L, L326I, P327T, V329T, and L481H. Reactions were carried out in 50 mM HEPES pH 8.0, 5 mM ATP, 5 mM $MgCl_2$, 0.05-0.50 μM enzyme, at 37 °C. Activity was measured by the rate of NAD(P)H oxidation (change in absorbance at 340 nm). Average of at least three independent experiments; ± indicates standard error.

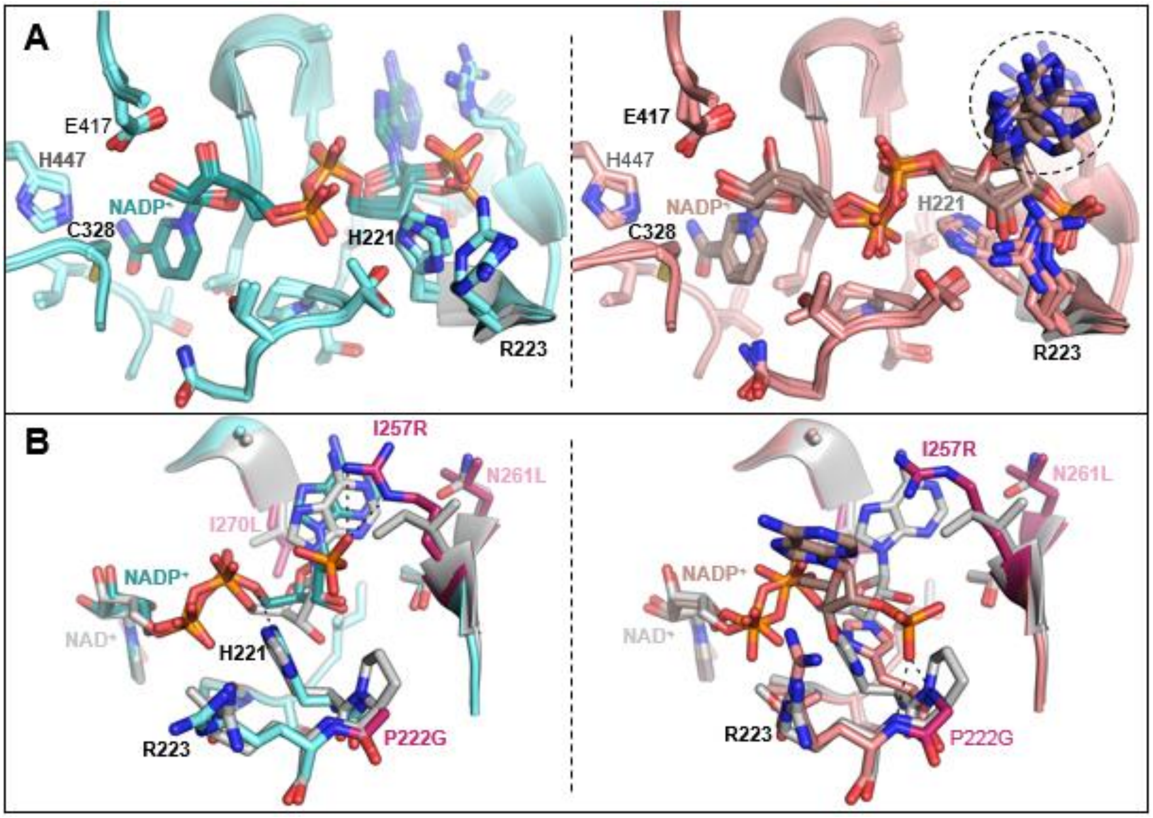

Figure S4. Superpositions of the NADP binding sites observed in the crystal structure of the engineered GCR (PDB 6GVS). A) Overlay of ‘canonical NADP binding mode’ observed in three out of 10 subunits of the GCR crystal structure (*left panel*; subunits A, G, I) compared with the ‘alternative NADP binding mode’ observed in 4 other subunits of the GCR crystal structure (right *panel*; subunits B,C,H,J). Note the varying orientations of the adenine ring (circled). **B)** Close-up on the adenosyl binding site superposing the crystal structures (RMSD of 0.276 Å over 424 Cα-atoms) of the engineered GCR with bound NADP (cyan) and the wild type enzyme (PduP) with bound NAD (PDB:5JFL; grey). The key mutations in the engineered GCR are highlighted in dark pink. In the canonical binding mode (cyan – left panel) the adenine ring of NADP assumes a similar position as for NAD in PduP (grey) and is held in place by a cation-π interaction with the introduced R257 (I257 in wild-type PduP), which is also coordinating the 2’-phophate group (dashed lines). In the alternative NADP binding mode (light pink – right panel), the adenine ring is pointing out of the binding pocket, although the 2’-phophate group is coordinated by the backbone of G222 and R223 (dashed lines). This mode is likley promoted by the P222G mutation. Note that H221 adopts different conformations in both binding modes. Nonetheless, the catalytically functional part of NADP, the nicotinamide moiety, assumes the same position in both modes and corresponds well to that of NAD in wild type PduP.

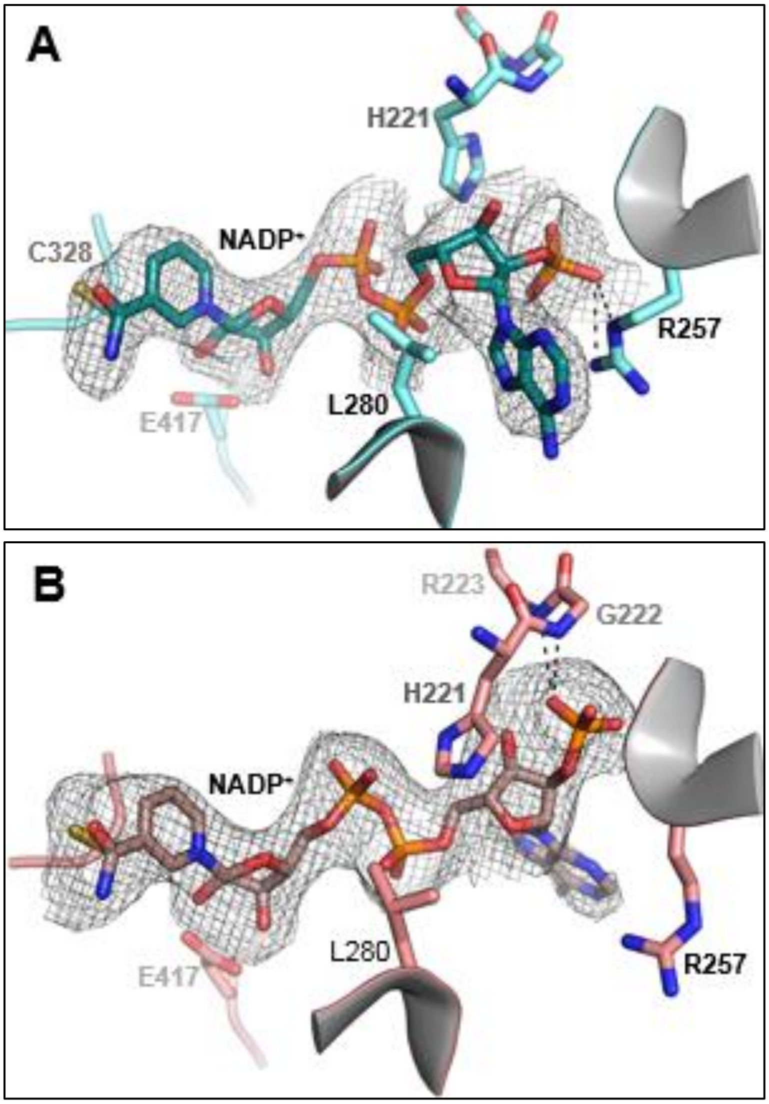

Figure S5. Simulated annealing Fo-Fc electron density maps of NADP binding. A) Omit map at 2.5 σ for NADP in ‘canonical binding mode’. The adenine ring is involved in a cation-π interaction with the introduced R257, and also coordinates the 2’-phophate group, as indicated by dashed lines. **B)** Omit map at 1.8 σ for NADP in ‘alternative binding mode’. The 2’-phosphate group is coordinated by interactions with the backbone of the introduced G222 and R223, indicated by dashes. The adenine ring is not coordinated resulting in flexibility and weak electron density.

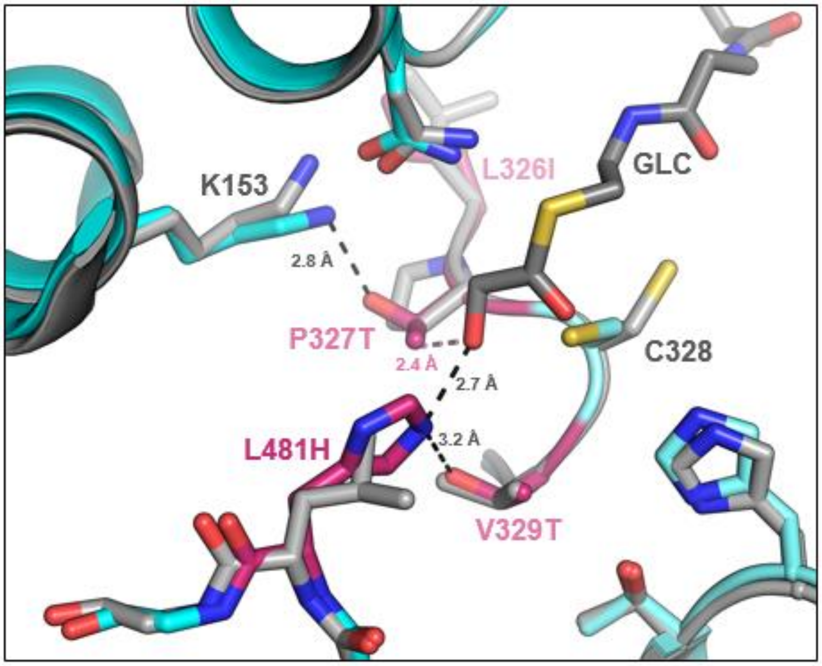

Figure S6. The putative glycolyl-CoA mode of binding in the engineered GCR and wild type PduP. The modeled glycolyl-CoA (GLC) in the engineered glycolyl-CoA reductase (cyan) is based on propionyl-CoA in the wild type PduP (PDB 5JFM; grey). Mutated residues are highlighted (dark pink). The terminal hydroxyl group of the glycolyl-CoA could participate in an H-bond with the introduced H481. In the GCR structure (apo-structure with respcct to glycolyl-CoA), the introduced T327 engages in an H-bond with K153. However in its alternative rotamer conformation, T327 would be in an ideal distance (pink dashed line) and angle to accept an H-bond from glycolyl-CoA. The RMSD for the superposition of the two structures is 0.551 Å over 417 Cα-atoms.

Supplemental Experimental Materials

Unless otherwise noted, all reagents were purchased from Merck/Sigma-Aldrich (Darmstadt, Germany). All enzymes for cloning were purchased from New England Biolabs (Ipswich, MA, USA).

Table S1. Materials used in this work.

Material	Supplier	Location
96-well deep plates	Axygen (Corning)	Corning, New York, US
Acetic Acid, glacial	Bio-lab	Jerusalem, Israel
Acetonitrile, ULC/MS-grade	Bio-lab	Jerusalem, Israel
Adenosine 5’-triphosphate disodium salt hydrate	Sigma (Merck)	Darmstadt Germany
Agarose	Hydragene	Hai Cang Indistrial Park, Xiamen, China
Amicon Ultra 0.5mL 10K centrifugal filters	Millipore/Merck	Darmstadt Germany
Ammonia solution, 25%	Merck	Darmstadt Germany
Ammonium acetate	Merck	Darmstadt Germany
Ampicillin Sodium Salt	MP (Fisher Scientific)	Hampton, NH, USA
Benzonase	Merck	Darmstadt Germany
Chloramphenicol	Sigma (Merck)	Darmstadt Germany
Coenzyme A	Ark Pharm Inc	Arlington Heights, IL, USA
DL-Dithiothreitol	Gold Biotechnology	St Louis, MO, USA
D-Glyceraldehyde 3-phosphate solution	Sigma (Merck)	Darmstadt Germany
5,5’-Dithiobis(2-nitrobenzoic acid)	Sigma (Merck)	Darmstadt Germany
Glucose	J.T. Baker (Fisher Scientific)	Hampton, NH, USA
Glycerol	J.T. Baker (Fisher Scientific)	Hampton, NH, USA
Glycolaldehyde dimer	Sigma (Merck)	Darmstadt Germany
Glycolic acid	Sigma (Merck)	Darmstadt Germany
HEPES	Apollo Scientific	Denton, Manchester
HisTrap™ FF column 5mL	GE Healthcare	Little Chalfont, UK
Hydrochloric acid	Bio-lab	Jerusalem, Israel
Imidazole	Glentham Life Science	Corsham, UK
IPTG	Inalco Pharmaceuticals	San Luis Obispo, CA, USA
Labcycler PCR Machine	Sensoquest	Göttingen, Germany
Liquid handling robot Precision 2000	BioTek	Winooski, Vermont, USA
L-Lactate dehydrogenase from rabbit muscle	Sigma (Merck)	Darmstadt Germany
Magnesium chloride	Sigma (Merck)	Darmstadt Germany
Methanol, ULC/MS-grade	Bio-lab	Jerusalem, Israel
Myokinase from rabbit muscle	Merck	Darmstadt Germany
Nickel-NTA beads	Adar Biotech	Rehovot, Israel
NAD+ sodium salt	Sigma (Merck)	Darmstadt Germany
NADH disodium salt	Calbiochem (Merck)	Darmstadt Germany
NADPH tetrasodium salt	Apollo Scientific	Bredbury, UK

Phosphoenolpyruvic acid monopotassium salt	Combi-blocks	Combi-blocks
PowerWave HT spectrophotometer	BioTek	Winooski, Vermont, USA
Propionic Acid	Combi-blocks	San Diego, CA
Protease inhibitor cocktail	Sigma (Merck)	Darmstadt Germany
Pyruvate kinase from rabbit muscle	Sigma (Merck)	Darmstadt Germany
Ribulose-1,5-bisphosphate sodium salt hydrate	Sigma (Merck)	Darmstadt Germany
SDS-PAGE gel, 12%	Expedeon	San Diego, CA, USA
Sodium chloride	Bio-lab	Jerusalem, Israel
Sodium hydroxide	Merck	Darmstadt Germany
Tris	Fisher Scientific	Hampton, NH, USA
UV-transparent 96-well microplate	Greiner	Kremsmünster, Austria
Water, ULC/MS-grade	Bio-lab	Jerusalem, Israel
Water, Molecular Biology Grade	Sigma (Merck)	Darmstadt Germany
Vibra-Cell sonicator	Sonics & Materials, Inc	Newton, CT, USA
Vivaspin 20 ultrafiltration spin tubes	GE Healthcare	Little Chalfont, UK

Supplemental Experimental Methods

Protein expression and purification

All enzymes described were His-tagged and purified on Ni-NTA beads. Isolated transformants were grown in 500 mL 2YT+0.1mg/mL ampicillin (pET vector) or 0.035 mg/mL chloramphenicol (ASKA vector) at 37 °C, 250 rpm, until an OD600 of 0.6. IPTG (0.4 mM) was used to induce gene expression at 16 °C, 250 rpm for 20 hours. The cells were harvested by centrifugation at 5,000g for 15 min at 4 °C, and the pellets were stored at -80 °C or directly used for protein purification.

Cell pellets were resuspended in 15 ml lysis buffer (50 mM HEPES pH 8, 50 mM NaCl, 2 mM $MgCl_2$, 0.1 mM DTT, 10 units/mL benzonase, 1:500 dilute Sigma protease inhibitor cocktail without EDTA) and sonicated. The supernatant was collected after centrifugation at 10,000 g at 4 °C for 30 min. His-tagged proteins were purified with nickel-NTA beads. After sample loading, columns were washed with 10 column volumes of wash buffer, and eluted in 10 mL of elution buffer (wash buffer was 50 mM HEPES pH 8, 300 mM sodium chloride, and 35 mM imidazole, and elution buffer was 50 mM HEPES pH 8, 50 mM sodium chloride, and 150 mM imidazole). The purified proteins were buffer-exchanged to 50 mM HEPES pH 8, 100 mM sodium chloride, 2 mM $MgCl_2$ using Vivaspin 20 ultrafiltration spin tubes, and their concentrations were calculated by their extinction coefficients at 280 nm. Proteins were stored at -20 °C with 10 % glycerol.

ACS deacylation with* E. coli *CobB

Prior to use, ACS preparations were treated with *E. coli* deacetylase CobB, expressed and purified from the ASKA collection. Deacetylation conditions were: 1 mM NAD^+, 50 mM HEPES pH 8, 100 mM NaCl, 2 mM $MgCl_2$, 20 μM ACS, 4 μM CobB, 37°C overnight.

E. coli CobB was expressed and purified from a pCA24N plasmid under a PT5-lac promoter in the following manner: Transformed BL21(DE3) cells were grown overnight on LB-agar plates with chloramphenicol (0.035 mg/L) + 0.1 % glucose. Freshly grown, isolated colonies were used to inoculate starter cultures in 2YT + chloramphenicol (35 mg/l) + 0.1 % glucose and grown at 37 °C overnight. Starters were then used to inoculate (1:50) cultures and grown at room temperature to OD600nm ≈ 1. Protein expression was induced by the addition of IPTG (final, 1 mM) to the growth cultures. Cultures were then grown at 16 °C for 24 h, after which cells were harvested and kept frozen at -80 °C until purification. Frozen cells were defrosted and lysed by re-suspension in 20 mM Tris-HCl pH 8, NaCl 250 mM, 0.4 mg/ml Lysozyme, 1 mM DTT, 1:500 dilute Sigma protease inhibitor cocktail without EDTA, 25 U/ml Benzonase Nuclease. The lysate was clarified by centrifugation (11,000 g, 4 °C, 30 min) and loaded on a 5ml HisTrap™ FF column pre-equilibrated with 20 mM Tris-HCl pH 8, 350 mM NaCl, 35 mM imidazole). Following an extensive (5 c.v) wash, the protein was eluted using 5 c.v of 20 mM Tris-HCl pH 8, 100 mM NaCl, 250 mM imidazole pH 8. Eluted fractions containing the purified CobB were pooled, dialyzed with 50 mM Tris-HCl pH 8, 100 mM NaCl, 20% Glycerol and kept frozen at -20 °C until use. Protein concentrations were examined by absorbance at 280 nm and by PAGE-gel densitometry using BSA for calibration.

Enzyme kinetics: Acyl-CoA Synthetase

A coupled assay was applied monitoring the conversion of ATP to AMP (1). Briefly, a 200 µL reaction mixture containing 50 mM HEPES pH8, 5 mM $MgCl_2$, 1 mM CoA, 1 mM DTT, 2.5 mM ATP, 0.6 mM NADH, 2.5 mM phospholenolpyruvate, 15 units/mL pyruvate kinase, 23 units/mL lactate dehydrogenase, 25 units/mL myokinase, 0.05-0.1 µM ACS enzyme, and various concentrations of carboxylic acids. Reactions were initiated by addition of the reaction mix to substrate in UV-transparent 96-well microplate and absorbance at 340 nm was monitored at 37 °C for 10 min.

We also applied an endpoint assay based on detection of unreacted CoA using 5,5'-Dithiobis(2-nitrobenzoic acid) (DTNB). 100 µL reactions were carried out with 0.05-0.1 µM enzyme, 250-500 µM CoA, 5 mM ATP, in 50mM HEPES pH 8, 5 mM $MgCl_2$, for 10 min at 37°C. Activity was measured by titrating the unreacted CoA using 100 µL of DTNB solution (2 mM DTNB, 50 mM HEPES pH 7.5) and absorbance was determined at 412 nm. There was qualitative agreement between the coupled assay and the endpoint assay.

Enzyme kinetics: Acyl-CoA reduction

The oxidation of $NAD(P)^+$ to NAD(P)H was monitored. Briefly, a 200 µL reaction mixture containing 50 mM HEPES pH 8.0, 5 mM $MgCl_2$, 1 mM DTT, and various concentrations of acyl-CoA and NAD(P)H was incubated with enzyme (0.05-0.5 µM) in UV-transparent 96-well microplate and absorbance at 340 nm was monitored at 37°C for 10 min. For combined assays (the reduction module), ACS variants, CoA, carboxylic acids, and ATP were added, and reduction was monitored by NADPH consumption.

Screening of mutant acetyl-CoA synthetase libraries

Plasmids encoding ACSstab library variants were transformed into electrocompetent *E. coli* BL21 (DE3) cells harboring a pCA24N plasmid with CobB from the ASKA collection. Individual transformants were picked and grown in LB-agar plates with ampicillin (0.1 mg/mL) + chloramphenicol (0.035 mg/mL) + 0.1% glucose. Overnight cultures were used to inoculate (1:100 dilution) fresh media (0.5 ml), grown at room temperature to OD600nm

≈ 0.6, induced with 1 mM IPTG, and grown at room temperature overnight. Cells were pelleted and lysed by resuspension in 300 µL/well of lysis buffer (50 mM HEPES pH 7.5, 0.1 M NaCl, 0.6 mg/ml lysozyme, 2 mM $MgCl_2$ and 25 U/mL benzonase) and shaking (1200 rpm) for 1h at 37°C. Lysates were pelleted by centrifugation and kept at 4°C before screening. Forty microliters of clear cell lysate from each well were transferred to a 96-well ELISA plate using an automated liquid handling robot and mixed with 60µL of a buffered glycolate solution (50 mM HEPES pH 7.5, 2 mM $MgCl_2$, 10 mM ATP, 0.5 mM CoA, 2 mM Glycolate) for 10 min at 37°C with shaking at 600 rpm. Unreacted CoA was detected with the DTNB assay. 100 µL of DTNB solution (2 mM DTNB, 50 mM HEPES pH 7.5) was added per well and absorbance was determined at 412 nm. Lysates of cells expressing ACSstab and lysis buffer itself, were used as negative controls.

Screening of mutant acyl-CoA reductase libraries

Libaries were cloned and transformed directly into electrocompetent *E. coli*BL21 (DE3) and plated on LB plates (0.1 mg/mL ampicillin). Individual transformants were picked and grown in 500 µL 2YT in 96-well deep-well plates at 37°C overnight. 100 µL of overnight cultures was transferred to a new 96-well deep-well plates and 400 µL of 2YT media with 0.1 mg/mL ampicillin were added and incubated at 37°C, 1000 rpm shaking for 4h. IPTG was added to 400 µM and the temperature was reduced to 16°C for 16 h. Cells were spun down and lysed with 300 µL/well lysis buffer (50 mM HEPES pH 8, 100 mM NaCl, 2 mM $MgCl_2$, 0.6 mg/mL lysozyme, 10 U/mL benzonase) at 37°C for 1h. After centrifugation, the clarified lysate (100µL) was added to 100µL reaction mixture (50mM HEPES pH 8, 5 mM $MgCl_2$, 1 mM DTT, various concentrations of acyl-CoA and NAD(P)H), and absorbance at 340 nm was monitored at 37°C for 10 min in UV-transparent 96-well plates. Lysates of cells expressing *Rp*PduP and lysis buffer itself were used as negative controls.

Thermostability assay of acetyl-Coenzyme A synthetases

ACS samples (0.1µM) were incubated at varied temperatures in a gradient PCR machine for 10min in 50uL volume, then cooled on ice and incubated with 500 µM acetate, 5 mM ATP, and 250 µM CoA in a total volume of 100 µL (50 mM HEPES pH 8, 5 mM $MgCl_2$), for 10 min at 37°C. Activity was detected with DTNB assay: To measure the concentration of unreacted CoA, 100 µL of 1 mM DTNB solution in 50 mM HEPES pH 8 was added and absorbance was measured at 412 nm in a PowerWave HT spectrophotometer (BioTek).

Chemical synthesis of glycolyl-CoA. Glycolyl-CoA was synthesized from glycolate and CoA using carbonyldiimidazole (CDI). 42.2 mg CDI was dissolved in 2 mL dry tetrahydrofuran (THF). 80 mg glycolate was added and incubated for 30 min at room temperature. CoA (80mg) was dissolved in 1 mL of 0.5 M $NaHCO_3$ pH 7.4, added to the stirred THF solution and incubated for 12 h on ice and resulting glycolyl-CoA was used for library screening or purified. For the latterthe reaction mixture was diluted with 9 mL of 0.5 M $NaHCO_3$ pH 7.4 and acidified to a pH of 3 by adding formic acid. The mixture was stirred and degassed under vaccum for 45 min at 4°C. The mixture was then applied to a preparative Agilent 1260 Infinity HPLC with a Phenomenex Gemini 10µm NX-C18 110 Å column. Chromatographic separation was achieved using 25 mM ammonium formate pH 4.2 with a methanol gradient at a flow rate of 25 mL min^{-1}: 0 min 1% methanol; 0.5 min 1% methanol, 10 min 30% methanol. Glycolyl-CoA fractions were pooled, flash-frozen in liquid nitrogen, and lyophilized. Glycolyl-CoA was approximately 90% pure as judged by HPLC. Purified glycolyl-CoA was used for kinetics.

Crystallization, and structure determination of the glycolyl-CoA reductase.

Heterologous expression of the glycolyl-CoA reductase - E. coli BL21 (DE3) cells containing the expression plasmid for the His_6-tagged GCR were grown in Terrific Broth.

Ampicillin was added to concentrations of 100 µg/ml. The cultures were grown at 37°C to an OD_{600nm} of 0.8 and induced with 0.25 mM IPTG. After induction the cultures were cooled to 25°C and grown overnight.

Purification, crystallization, and structure determination of the glycolyl-CoA reductase - Cells that expressed *GCR* were resuspended in a 3-fold volume of 50 mM MOPS/KOH buffer (pH 7.5) containing 250 mM KCl (buffer A) with 0.1 mg DNase I per mL. The cells were lysed by passage through a microfluidizer (LM10, Microfluidics Corp., USA) at 110 MPa. The cell lysates were ultra centrifuged for 1 h at 100,000×*g* at 4°C and the supernatants were applied at a flow rate of 1 mL min^{-1} to a 1 mL HisTrap FF column (GE Healthcare) that had been equilibrated with buffer A. The column was washed with 60 mM imidazole in buffer A and GCR was eluted with 500 mM imidazole in buffer A. Fractions containing GCR were pooled and concentrated using a Amicon Ultra-4 Centrifugal Filter Unit (EMD Millipore) with a 100 kDa pore size. The sample was then applied to a 120 mL gel filtration column (HiLoad 16/600 Superdex 200 pg, GE Healthcare), equilibrated with 20 mM MOPS/KOH pH 7.5 buffer containing 100 mM KCl. The protein was kept on ice over night before crystal plates where set up.

For co-crystallization of the purified His_6-tagged GCR in the presence of $NADP^+$ the enzyme (6.5 mg mL^{-1}) was mixed with 0.2 M potassium citrate/KOH pH 8.4, 20% (w/v) PEG 3350, and supplemented with $NADP^+$ from a 30 mM stock in a ratio of 2µL: 2µL: 1µL (enzyme: crystallization buffer: substrate). Thick needle shaped crystals appeared overnight and the over the course of two days. Crystals were soaked briefly with mother liquor supplemented with 30 % (v/v) ethylene glycol, and 10 mM $NADP^+$, before freezing in liquid nitrogen. X-ray diffraction data were collected at the beamline ID30B of the ESRF (Grenoble, France) and at the beamlines P13 of the DESY (Hamburg, Germany).

The data was processed with the XDS software package(2). The structures were solved by molecular replacement using the structure of the wild type propionaldehyde dehydrogenase from *Rhodopseudomonas palustris* (3) (PDB 5JFL) as the search model. The molecular replacement was carried out using Phaser of the Phenix software package(4) and refined with Phenix.Refine. Additional modeling, manual refining and ligand fitting was done in COOT(5). Final positional and B-factor refinements, as well as water-picking for the structure were performed using Phenix.Refine. The GCR structure model was deposited at the Protein Bata Bank in Europe (PDBe) under the PDB ID 6GVS.

Table S2. X-ray data collection and refinement statistics for the GCR structure

Beam line	ID30B ESRF, Grenoble, France
PDB ID	6GVS
Ligands	$NADP^+$, K^+
Wavelength	0.9762
Resolution range (Å)	39.13 - 2.58 (2.72 - 2.58)
Space group	C 1 2 1
Unit cell dimensions	
a b c (Å)	362.20 123.18 165.10
α β γ (°)	90.00 109.25 90.00
Total reflections	853804 (123635)
Unique reflections	212592 (30472)
Multiplicity	4.6 (4.7)
Completeness (%)	98.9 (97.8)
Mean $I/\sigma(I)$	7.6 (1.5)
R_{merge}	0.130 (0.969)
R_{pim}	0.074 (0.548)
$CC_{1/2}$	0.993 (0.598)
**Refinement**	
R_{work} / R_{free}	0.1915 / 0.2098
Number of non-hydrogen atoms	33944
macromolecules	32970
ligands	470
solvent	504
RMS(bonds)	0.009
RMS(angles)	0.724
Ramachandran	
favored (%)	97.79
allowed (%)	2.21
outliers (%)	0.00
Average B-factor	58.08
macromolecules	57.71
ligands	86.68
solvent	55.06

Values in parentheses are for highest-resolution shell.

LC-MS pathway analysis. 100 µL reaction mix was cleared of protein using Amicon Ultra 0.5 mL 10K centrifugal filters. Samples were run on a Waters e2695 Separation modue with a Phenomenex Luna-NH_2 column (3µm, 100 Å) and detected on a Waters Acquity QDA system. Chromatographic separation used the following gradient conditions at a flow rate of 0.25 mL/min (acetonitrile = A, 10 mM ammonium acetate pH 10 = B) : 0 min 85% A, 15% B; 7 min 5% A, 95% B; 14 min 5% A, 95% B; 15 min 85% A 15% B; 17 min 85% A 15% B. Ribulose-1,5-bisphosphate (>99.0% purity) was used as a standard.

Table S3. Primers used in this work.

Primer name	Sequence	Purpose
T7SeqFWD	TAATACGACTCACTATAGGG	General sequencing primer for pET vector
T7SeqREV	GCTAGTTATTGCTCAGCGG	General sequencing primer for pET vector
AdaptorFWD	GACGAGGTGATCAACACCCAT	Adaptor sequence for synthesized genes
AdaptorREV	ATCAGCGACGAGAGCACCCTCGAG	Adaptor sequence for synthesized genes
NADPHlib-1FWD	CAGCCCGCATNSACGCGCCC	Mutagenic primer for PduP NADPH switch
NADPHlib-1REV	GGGCGGGTCNATGCGGGCTG	Mutagenic primer for PduP NADPH switch
NADPHlib-2FWD	GAAGCCCTCGANAGAGAACRMCAACGCCATG	Mutagenic primer for PduP NADPH switch
NADPHlib-2REV	CATGGCGTTGKYGTTCTCTNTCGAGGGCTTC	Mutagenic primer for PduP NADPH switch
NADPH.N263XFWD	CACCNNKGCCATGATGGCGC	Mutagenic primer for PduP NADPH switch
NADPH.N263XREV	GCGCCATCATGGCMNNGGTG	Mutagenic primer for PduP NADPH switch
NADPH.I282XFWD	CGGCCCCGCCNNKGTCAAGGCG	Mutagenic primer for PduP NADPH switch
NADPH.I282XREV	CGCCTTGACMNNGGCGGGGCCG	Mutagenic primer for PduP NADPH switch
PduPglycFWD	CTTCGACAACAACVTRSHRTGCRYTGCCGAGAAGGAG	Mutagenic primer for PduP Glycolyl-CoA activity
PduPglycREV	CTCCTTCTCGGCARYGCAYDSYABGTTGTTGTCGAAG	Mutagenic primer for PduP Glycolyl-CoA activity

PduPglycL483FWD	CTTATGCGGGGNNSGGCGCCGGCGGCG	Mutagenic primer for PduP Glycolyl-CoA activity
PduPglycL483REV	CGCCGCCGGCGCCSNNCCCCGCATAAG	Mutagenic primer for PduP Glycolyl-CoA activity
PduPglycA298FWD	GATCGGCGCCGGCNNSGGCAATCCGCCCG	Mutagenic primer for PduP Glycolyl-CoA activity
PduPglycA298REV	CGGGCGGATTGCCSNNGCCGGCGCCGATC	Mutagenic primer for PduP Glycolyl-CoA activity
PduPglycN199FWD	CGCCGACCACCNNSCCGACGGAAACC	Mutagenic primer for PduP Glycolyl-CoA activity
PduPglycN199REV	GGTTTCCGTCGGSNNGGTGGTCGGCG	Mutagenic primer for PduP Glycolyl-CoA activity

Gene and amino acid sequences

>*E. coli* ACS

ASKA clone JW4030

>*E. coli* FsaA

ASKA clone JW5109

>*E. coli* FsaB

ASKA clone JW3918

>*E. coli* KdsD

ASKA clone JW3164

>*E. coli* GutQ

ASKA clone JW5431

>Rhodobacter sphaeroides Phosphoribulokinase DNA

ATGAGCAAGAAGCATCCCATCATCTCGGTCACGGGCTCGTCCGGCGCGGGCACCTCGACGGTGAA
GCACACGTTCGACCAGATCTTCCGGCGCGAGGGGGTCAAGGCGGTCTCGATCGAGGGCGACGCCT
TCCACCGCTTCAACCGCGCCGACATGAAGGCCGAGCTGGACCGGCGCTATGCGGCGGGCGACGCC
ACCTTCTCGCACTTCTCCTACGAGGCGAACGAGCTGAAGGAGCTGGAGCGCGTCTTCCGCGAGTA
CGGCGAGACCGGGCAGGGGCGGACGCGGACCTACGTCCATGACGATGCCGAAGCGGCGCGCACCG
GCGTGGCGCCCGGCAACTTCACCGACTGGCGCGACTTCGACAGCGACAGCCACCTGCTGTTCTAC
GAGGGGTTGCACGGGGCGGTGGTGAACAGCGAGGTCAATATCGCGGGGCTGGCCGACCTCAAGAT
CGGCGTGGTTCCGGTCATCAACCTCGAATGGATCCAGAAGATCCACCGCGACCGCGCGACCCGCG
GTTACACGACCGAGGCGGTCACCGACGTGATCCTGCGCCGGATGCATGCCTACGTTCACTGCATC
GTGCCGCAATTCTCGCAGACCGACATCAACTTCCAGCGCGTGCCGGTGGTGGATACCTCGAACCC
CTTCATCGCGCGCTGGATCCCCACCGCCGACGAGAGCGTGGTGGTGATCCGTTTCCGCAATCCGC
GCGGGATCGACTTCCCCTATCTCACCTCGATGATCCACGGCTCGTGGATGAGCCGCGCCAATTCC

ATCGTGGTGCCCGGCAACAAGCTGGACCTCGCGATGCAGCTGATCCTGACACCGCTGATCGACCG
GGTCGTCCGAGAGAGCAAGGTGGCCCTCGAGCACCACCACCACCACCACTGA

>Rhodobacter sphaeroides Phosphoribulokinase AA

MSKKHPIISVTGSSGAGTSTVKHTFDQIFRREGVKAVSIEGDAFHRFNRADMKAELDRRYAAGDA
TFSHFSYEANELKELERVFREYGETGQGRTRTYVHDDAEAARTGVAPGNFTDWRDFDSDSHLLFY
EGLHGAVVNSEVNIAGLADLKIGVVPVINLEWIQKIHRDRATRGYTTEAVTDVILRRMHAYVHCI
VPQFSQTDINFQRVPVVDTSNPFIARWIPTADESVVVIRFRNPRGIDFPYLTSMIHGSWMSRANS
IVVPGNKLDLAMQLILTPLIDRVVRESKVALEHHHHHH*

>ACSstab DNA

ATGGCTCAGACCCACAAACACCCGGTTCCGCCGAACATCGCGGCGAACGCGTGGATCAACCCGGA
ACAGTACGAAGCGATGTACAAACAGTCTATCAACGACCCGGACACCTTCTGGCGTGAACAGGGTA
AAATCCTGGACTGGATCAAACCGTACACCCGTGTTAAAAACACCTCTTTCGAACCGGGTAACGTT
TCTATCCGTTGGTACGAAGACGGTACCCTGAACCTGGCGTACAACTGCCTGGACCGTCACCTGGA
AGAACGTGGTGACCAGACCGCGATCATCTGGGAAGGTGACGACCCGTCTGAATCTCGTCACATCA
CCTACCGTGAACTGCACCGTGACGTTTGCCGTTTCGCGAACGTTCTGAAAGCGCTGGGTATCAAA
AAAGGTGACGTTGTTGCGATCTACATGCCGATGGTTCCGGAAGCGGCGGTTGCGATGCTGGCGTG
CGCGCGTATCGGTGCGATCCACTCTGTTATCTTCGGTGGTTTCTCTCCGGAAGCGGTTGCGGGTC
GTATCATCGACTCTAACTCTCGTCTGGTTATCACCGCGGACGAAGGTGTTCGTGCGGGTCGTTCT
ATCCCGCTGAAAAAGAACGTTGACGAAGCGCTGAAAAACCCGAACGTTACCTCTGTTGAACACGT
TATCGTTTTCAAACGTACCGGTGGTGACATCGACTGGCAGGAAGGTCGTGACCTGTGGTGGCGTG
ACCTGATCGAAAAAGCGTCTCCGGAACACCAGCCGGAACCGATGAACGCGGAAGACCCGCTGTTC
ATCCTGTACACCTCTGGTTCTACCGGTAAACCGAAAGGTGTTCTGCACACCACCGGTGGTTACCT
GGTTTACGCGGCGACCACCTTCAAATACGTTTTCGACTACCACCCGGGTGACATCTACTGGTGCA
CCGCGGACGTTGGTTGGGTTACCGGTCACTCTTACCTGCTGTACGGTCCGCTGGCGTGCGGTGCG
ACCACCCTGATGTTCGAAGGTGTTCCGAACTGGCCGACCCCGTCTCGTATGGCGCAGGTTGTTGA
CAAACACCAGGTTAACATCCTGTACACCGCGCCGACCGCGATCCGTGCGCTGATGGCGGAAGGTG
ACAAAGCGATCGAAGGTACCGACCGTTCTTCTCTGCGTATCCTGGGTTCTGTTGGTGAACCGATC
AACCCGGAAGCGTGGGAATGGTACTGGAAAAAAATCGGTAACGAACGTTGCCCGGTTGTTGACAC
CTGGTGGCAGACCGAAACCGGTGGTTTCATGATCACCCCGCTGCCGGGTGCGATCGAACTGAAAC
CGGGTTCTGCGACCCGTCCGTTCTTCGGTGTTCAGCCGGCGCTGGTTGACAACGAAGGTAACCCG
CAGGAAGGTGCGACCGAAGGTAACCTGGTTATCACCGACTCTTGGCCGGGTCAGGCGCGTACCCT
GTACGGTGACCACGAACGTTTCGAACAGACCTACTTCTCTACCTTCAAAGGTATGTACTTCTCTG
GTGACGGTGCGCGTCGTGACGAAGACGGTTACTACTGGATCACCGGTCGTGTTGACGACGTTCTG
AACGTTTCTGGTCACCGTCTGGGTACCGCGGAAATCGAATCTGCGCTGGTTGCGCACCCGAAAAT
CGCGGAAGCGGCGGTTGTTGGTATCCCGCACGACATCAAAGGTCAGGCGATCTACGCGTACGTTA
CCCTGAACCACGGTGAAGAACCGTCTCCGGAACTGTACAAAGAAGTTCGTAACTGGGTTCGTAAA
GAAATCGGTCCGCTGGCGACCCCGGACGTTCTGCACTGGACCGACGGTCTGCCGAAAACCCGTTC
TGGTAAAATCATGCGTCGTATCCTGCGTAAAATCGCGGCGGGTGACACCTCTAACCTGGGTGACA
CCTCTACCCTGGCGGACCCGTCTGTTGTTGACAAACTGATCGAAGAAAAACAGGCGATCGCGATG
CCGTCTCTCGAGCACCACCACCACCACCACTGA

>ACSstab AA

MAQTHKHPVPPNIAANAWINPEQYEAMYKQSINDPDTFWREQGKILDWIKPYTRVKNTSFEPGNV
SIRWYEDGTLNLAYNCLDRHLEERGDQTAIIWEGDDPSESRHITYRELHRDVCRFANVLKALGIK
KGDVVAIYMPMVPEAAVAMLACARIGAIHSVIFGGFSPEAVAGRIIDSNSRLVITADEGVRAGRS
IPLKKNVDEALKNPNVTSVEHVIVFKRTGGDIDWQEGRDLWWRDLIEKASPEHQPEPMNAEDPLF
ILYTSGSTGKPKGVLHTTGGYLVYAATTFKYVFDYHPGDIYWCTADVGWVTGHSYLLYGPLACGA
TTLMFEGVPNWPTPSRMAQVVDKHQVNILYTAPTAIRALMAEGDKAIEGTDRSSLRILGSVGEPI
NPEAWEWYWKKIGNERCPVVDTWWQTETGGFMITPLPGAIELKPGSATRPFFGVQPALVDNEGNP

QEGATEGNLVITDSWPGQARTLYGDHERFEQTYFSTFKGMYFSGDGARRDEDGYYWITGRVDDVL
NVSGHRLGTAEIESALVAHPKIAEAAVVGIPHDIKGQAIYAYVTLNHGEEPSPELYKEVRNWVRK
EIGPLATPDVLHWTDGLPKTRSGKIMRRILRKIAAGDTSNLGDTSTLADPSVVDKLIEEKQAIAM
PSLEHHHHHH*

>ACS19 DNA

ATGGCTCAGACCCACAAACACCCGGTTCCGCCGAACATCGCGGCGAACGCGTGGATCAACCCGGA
ACAGTACGAAGCGATGTACAAACAGTCTATCAACGACCCGGACACCTTCTGGCGTGAACAGGGTA
AAATCCTGGACTGGATCAAACCGTACACCCGTGTTAAAAACACCTCTTTCGAACCGGGTAACGTT
TCTATCCGTTGGTACGAAGACGGTACCCTGAACCTGGCGTACAACTGCCTGGACCGTCACCTGGA
AGAACGTGGTGACCAGACCGCGATCATCTGGGAAGGTGACGACCCGTCTGAATCTCGTCACATCA
CCTACCGTGAACTGCACCGTGACGTTTGCCGTTTCGCGAACGTTCTGAAAGCGCTGGGTATCAAA
AAAGGTGACGTTGTTGCGATCTACATGCCGATGGTTCCGGAAGCGGCGGTTGCGATGCTGGCGTG
CGCGCGTATCGGTGCGATCCACTCTGTTATCTTCGGTGGTTTCTCTCCGGAAGCGGTTGCGGGTC
GTATCATCGACTCTAACTCTCGTCTGGTTATCACCGCGGACGAAGGTGTTCGTGCGGGTCGTTCT
ATCCCGCTGAAAAAGAACGTTGACGAAGCGCTGAAAAACCCGAACGTTACCTCTGTTGAACACGT
TATCGTTTTCAAACGTACCGGTGGTGACATCGACTGGCAGGAAGGTCGTGACCTGTGGTGGCGTG
ACCTGATCGAAAAAGCGTCTCCGGAACACCAGCCGGAACCGATGAACGCGGAAGACCCGCTGTTC
ATCCTGTACACCTCTGGTTCTACCGGTAAACCGAAAGGTGTTCTGCACACCACCGGTGGTTACCT
GGTTTACGCGGCGACCACCTTCAAATACGTTTTCGACTACCACCCGGGTGACATCTACTGGTGCA
CCGCGGACGTTGGTTGGATTACCGGTCACGCGTATCTGCTGTACGGTCCGCTGGCGTGCGGTGCG
ACCACCCTGATGTTCGAAGGTGTTCCGAACTGGCCGACCCCGTCTCGTATGGCGCAGGTTGTTGA
CAAACACCAGGTTAACATCCTGTTTACCGCGCCGACCGCGATCCGTGCGCTGATGGCGGAAGGTG
ACAAAGCGATCGAAGGTACCGACCGTTCTTCTCTGCGTATCCTGGGTTCTACCGGTGAACCGATC
AACCCGGAAGCGTGGGAATGGTACTGGAAAAAAATCGGTAACGAACGTTGCCCGGTTGTTGACAC
CTGGTGGCAGACCGAAACCGGTGGTTGCATGATCACCCCGCTGCCGGGTGCGATCGAACTGAAAC
CGGGTTCTGCGACCCGTCCGTTCTTCGGTGTTCAGCCGGCGCTGGTTGACAACGAAGGTAACCCG
CAGGAAGGTGCGACCGAAGGTAACCTGGTTATCACCGACTCTTGGCCGGGTCAGGCGCGTACCCT
GTACGGTGACCACGAACGTTTCGAACAGACCTACTTCTCTACCTTCAAAGGTATGTACTTCTCTG
GTGACGGTGCGCGTCGTGACGAAGACGGTTACTACTGGATCACCGGTCGTGTTGACGACGTTCTG
AACGTTTCTGGTCACCGTCTGGGTACCGCGGAAATCGAATCTGCGCTGGTTGCGCACCCGAAAAT
CGCGGAAGCGGCGGTTGTTGGTATCCCGCACGACATCAAAGGTCAGGCGATCTACGCGTACGTTA
CCCTGAACCACGGTGAAGAACCGTCTCCGGAACTGTACAAAGAAGTTCGTAACTGGGTTCGTAAA
GAAATCGGTCCGCTGGCGACCCCGGACGTTCTGCACTGGACCGACGGTCTGCCGAAAACCCGTTC
TGGTAAAATCATGCGTCGTATCCTGCGTAAAATCGCGGCGGGTGACACCTCTAACCTGGGTGACA
CCTCTACCCTGGCGGACCCGTCTGTTGTTGACAAACCGATCGAAGAAAAACAGGCGATCGCGATG
CCGTCTCTCGAGCACCACCACCACCACCACTGA

>ACS19 AA

MAQTHKHPVPPNIAANAWINPEQYEAMYKQSINDPDTFWREQGKILDWIKPYTRVKNTSFEPGNV
SIRWYEDGTLNLAYNCLDRHLEERGDQTAIIWEGDDPSESRHITYRELHRDVCRFANVLKALGIK
KGDVVAIYMPMVPEAAVAMLACARIGAIHSVIFGGFSPEAVAGRIIDSNSRLVITADEGVRAGRS
IPLKKNVDEALKNPNVTSVEHVIVFKRTGGDIDWQEGRDLWWRDLIEKASPEHQPEPMNAEDPLF
ILYTSGSTGKPKGVLHTTGGYLVYAATTFKYVFDYHPGDIYWCTADVGWITGHAYLLYGPLACGA
TTLMFEGVPNWPTPSRMAQVVDKHQVNILFTAPTAIRALMAEGDKAIEGTDRSSLRILGSTGEPI
NPEAWEWYWKKIGNERCPVVDTWWQTETGGCMITPLPGAIELKPGSATRPFFGVQPALVDNEGNP
QEGATEGNLVITDSWPGQARTLYGDHERFEQTYFSTFKGMYFSGDGARRDEDGYYWITGRVDDVL
NVSGHRLGTAEIESALVAHPKIAEAAVVGIPHDIKGQAIYAYVTLNHGEEPSPELYKEVRNWVRK
EIGPLATPDVLHWTDGLPKTRSGKIMRRILRKIAAGDTSNLGDTSTLADPSVVDKPIEEKQAIAM
PSLEHHHHHH*

>RpPduP DNA

ATGGCACATCACCACCACCATCACGTGGGTACCAATGACGCAAACATCGCCGATGTCGTGACGAA
GGTCCTCGGCGAATATGGCGCGCCTGGTGCGGTGTCGGTCGCGGCGCTGACCGCCAAGAGCCCCG
ACGGGAAAAGCAACTCGTCCGCCGATGCGGATGTGGTCGCCCGGATGGTGGCCAAGGCGATCCGC
GACCACGCCGGAACTGCGCAGCCGTCCGGTAACGCCGCGACGTCGTCGGCGGCGGTCAGCGATGG
CGTGTTCGAGACCATGGATGCCGCGGTCGAGGCCGCTGCTCTGGCGCAGCAGCAATACCTGCTTT
GCTCGATGTCCGACCGCGCGCGCTTCGTGCAGGGAATCCGCGACGTCATCTTGAATCAGGATACG
CTCGAAAAGATGTCCCGCATGGCGGTCGAAGAAACCGGCATGGGCAATTACGAGCATAAGCTGAT
CAAGAACCGGCTAGCCGGCGAAAAGACCCCGGGCATCGAAGATCTCACCACCGACGCTTTCAGCG
GCGACAACGGTCTGACGCTGGTGGAATACTCGCCGTTCGGGGTGATCGGCGCGATCACGCCGACC
ACCAATCCGACGGAAACCATCGTCTGCAATTCGATCGGAATGCTGGCGGCCGGCAACAGCGTGGT
GTTCAGCCCGCATCCGCGCGCCCGGCAGGTGTCGCTGCTGCTGGTCCGCCTGATCAACCAGAAGC
TGGCAGCGCTCGGCGCGCCGGAAAACCTGGTGGTGACCGTGGAGAAGCCCTCGATCGAGAACACC
AACGCCATGATGGCGCATCCCAAGGTGCGGATGCTGGTTGCCACCGGCGGCCCCGCCATCGTCAA
GGCGGTGCTGTCGACCGGCAAGAAGGCGATCGGCGCCGGCGCCGGCAATCCGCCCGTCGTGGTCG
ACGAAACCGCCAACATCGAAAAAGCCGCCTGCGACATCGTCAATGGCTGCAGCTTCGACAACAAC
CTGCCCTGCGTCGCCGAGAAGGAGATCATCGCAGTCGCCCAGATCGCCGATTACCTGATCTTCAA
CTTGAAGAAGAACGGCGCCTACGAGATCAAGGATCCGGCGGTTCTGCAGCAACTGCAGGACCTGG
TGCTGACCGCCAAGGGCGGGCCACAGACCAAATGCGTCGGCAAGAGTGCGGTGTGGCTGCTGTCG
CAGATCGGCATCAGCGTCGACGCCAGCATCAAGATCATCCTGATGGAAGTGCCGCGGGAACATCC
CTTCGTGCAGGAAGAACTGATGATGCCGATCCTGCCGCTGGTCCGGGTCGAAACCGTGGATGATG
CGATCGACCTCGCCATCGAGGTGGAGCACGACAATCGCCACACCGCGATCATGCATTCCACCGAT
GTGCGCAAGCTCACCAAGATGGCCAAGCTGATCCAGACCACGATCTTCGTGAAGAACGGCCCGTC
TTATGCGGGGCTCGGCGCCGGCGGCGAGGGATATTCCACCTTCACCATCGCGGGTCCCACGGGCG
AGGGCCTGACCTCGGCGAAGTCGTTCGCGCGCCGCCGCAAATGCGTGATGGTGGAAGCGCTCAAT
ATCCGTTGA

>RpPduP AA

MAHHHHHHVGTNDANIADVVTKVLGEYGAPGAVSVAALTAKSPDGKSNSSADADVVARMVAKAIR
DHAGTAQPSGNAATSSAAVSDGVFETMDAAVEAAALAQQQYLLCSMSDRARFVQGIRDVILNQDT
LEKMSRMAVEETGMGNYEHKLIKNRLAGEKTPGIEDLTTDAFSGDNGLTLVEYSPFGVIGAITPT
TNPTETIVCNSIGMLAAGNSVVFSPHPRARQVSLLLVRLINQKLAALGAPENLVVTVEKPSIENT
NAMMAHPKVRMLVATGGPAIVKAVLSTGKKAIGAGAGNPPVVVDETANIEKAACDIVNGCSFDNN
LPCVAEKEIIAVAQIADYLIFNLKKNGAYEIKDPAVLQQLQDLVLTAKGGPQTKCVGKSAVWLLS
QIGISVDASIKIILMEVPREHPFVQEELMMPILPLVRVETVDDAIDLAIEVEHDNRHTAIMHSTD
VRKLTKMAKLIQTTIFVKNGPSYAGLGAGGEGYSTFTIAGPTGEGLTSAKSFARRRKCVMVEALN
IR

>Pdup-NP DNA

ATGGCACATCACCACCACCATCACGTGGGTACCAATGACGCAAACATCGCCGATGTCGTGACGAA
GGTCCTCGGCGAATATGGCGCGCCTGGTGCGGTGTCGGTCGCGGCGCTGACCGCCAAGAGCCCCG
ACGGGAAAAGCAACTCGTCCGCCGATGCGGATGTGGTCGCCCGGATGGTGGCCAAGGCGATCCGC
GACCACGCCGGAACTGCGCAGCCGTCCGGTAACGCCGCGACGTCGTCGGCGGCGGTCAGCGATGG
CGTGTTCGAGACCATGGATGCCGCGGTCGAGGCCGCTGCTCTGGCGCAGCAGCAATACCTGCTTT
GCTCGATGTCCGACCGCGCGCGCTTCGTGCAGGGAATCCGCGACGTCATCTTGAATCAGGATACG
CTCGAAAAGATGTCCCGCATGGCGGTCGAAGAAACCGGCATGGGCAATTACGAGCATAAGCTGAT
CAAGAACCGGCTAGCCGGCGAAAAGACCCCGGGCATCGAAGATCTCACCACCGACGCTTTCAGCG
GCGACAACGGTCTGACGCTGGTGGAATACTCGCCGTTCGGGGTGATCGGCGCGATCACGCCGACC
ACCAATCCGACGGAAACCATCGTCTGCAATTCGATCGGAATGCTGGCGGCCGGCAACAGCGTGGT
GTTCAGCCCGCATGGACGCGCCCGGCAGGTGTCGCTGCTGCTGGTCCGCCTGATCAACCAGAAGC

TGGCAGCGCTCGGCGCGCCGGAAAACCTGGTGGTGACCGTGGAGAAGCCCTCGAGAGAGAACACC
CTTGCCATGATGGCGCATCCCAAGGTGCGGATGCTGGTTGCCACCGGCGGCCCCGCCCTCGTCAA
GGCGGTGCTGTCGACCGGCAAGAAGGCGATCGGCGCCGGCGCCGGCAATCCGCCCGTCGTGGTCG
ACGAAACCGCCAACATCGAAAAAGCCGCCTGCGACATCGTCAATGGCTGCAGCTTCGACAACAAC
CTGCCCTGCGTCGCCGAGAAGGAGATCATCGCAGTCGCCCAGATCGCCGATTACCTGATCTTCAA
CTTGAAGAAGAACGGCGCCTACGAGATCAAGGATCCGGCGGTTCTGCAGCAACTGCAGGACCTGG
TGCTGACCGCCAAGGGCGGGCCACAGACCAAATGCGTCGGCAAGAGTGCGGTGTGGCTGCTGTCG
CAGATCGGCATCAGCGTCGACGCCAGCATCAAGATCATCCTGATGGAAGTGCCGCGGGAACATCC
CTTCGTGCAGGAAGAACTGATGATGCCGATCCTGCCGCTGGTCCGGGTCGAAACCGTGGATGATG
CGATCGACCTCGCCATCGAGGTGGAGCACGACAATCGCCACACCGCGATCATGCATTCCACCGAT
GTGCGCAAGCTCACCAAGATGGCCAAGCTGATCCAGACCACGATCTTCGTGAAGAACGGCCCGTC
TTATGCGGGGCTCGGCGCCGGCGGCGAGGGATATTCCACCTTCACCATCGCGGGTCCCACGGGCG
AGGGCCTGACCTCGGCGAAGTCGTTCGCGCGCCGCCGCAAATGCGTGATGGTGGAAGCGCTCAAT
ATCCGTTGA

>Pdup-NP AA

MAHHHHHHVGTNDANIADVVTKVLGEYGAPGAVSVAALTAKSPDGKSNSSADADVVARMVAKAIR
DHAGTAQPSGNAATSSAAVSDGVFETMDAAVEAAALAQQQYLLCSMSDRARFVQGIRDVILNQDT
LEKMSRMAVEETGMGNYEHKLIKNRLAGEKTPGIEDLTTDAFSGDNGLTLVEYSPFGVIGAITPT
TNPTETIVCNSIGMLAAGNSVVFSPHGRARQVSLLLVRLINQKLAALGAPENLVVTVEKPSRENT
LAMMAHPKVRMLVATGGPALVKAVLSTGKKAIGAGAGNPPVVVDETANIEKAACDIVNGCSFDNN
LPCVAEKEIIAVAQIADYLIFNLKKNGAYEIKDPAVLQQLQDLVLTAKGGPQTKCVGKSAVWLLS
QIGISVDASIKIILMEVPREHPFVQEELMMPILPLVRVETVDDAIDLAIEVEHDNRHTAIMHSTD
VRKLTKMAKLIQTTIFVKNGPSYAGLGAGGEGYSTFTIAGPTGEGLTSAKSFARRRKCVMVEALN
IR

>GCR DNA

ATGGCACATCACCACCACCATCACGTGGGTACCAATGACGCAAACATCGCCGATGTCGTGACGAA
GGTCCTCGGCGAATATGGCGCGCCTGGTGCGGTGTCGGTCGCGGCGCTGACCGCCAAGAGCCCCG
ACGGGAAAAGCAACTCGTCCGCCGATGCGGATGTGGTCGCCCGGATGGTGGCCAAGGCGATCCGC
GACCACGCCGGAACTGCGCAGCCGTCCGGTAACGCCGCGACGTCGTCGGCGGCGGTCAGCGATGG
CGTGTTCGAGACCATGGATGCCGCGGTCGAGGCCGCTGCTCTGGCGCAGCAGCAATACCTGCTTT
GCTCGATGTCCGACCGCGCGCGCTTCGTGCAGGGAATCCGCGACGTCATCTTGAATCAGGATACG
CTCGAAAAGATGTCCCGCATGGCGGTCGAAGAAACCGGCATGGGCAATTACGAGCATAAGCTGAT
CAAGAACCGGCTAGCCGGCGAAAAGACCCCGGGCATCGAAGATCTCACCACCGACGCTTTCAGCG
GCGACAACGGTCTGACGCTGGTGGAATACTCGCCGTTCGGGGTGATCGGCGCGATCACGCCGACC
ACCAATCCGACGGAAACCATCGTCTGCAATTCGATCGGAATGCTGGCGGCCGGCAACAGCGTGGT
GTTCAGCCCGCATGGACGCGCCCGGCAGGTGTCGCTGCTGCTGGTCCGCCTGATCAACCAGAAGC
TGGCAGCGCTCGGCGCGCCGGAAAACCTGGTGGTGACCGTGGAGAAGCCCTCGAGAGAGAACACC
CTTGCCATGATGGCGCATCCCAAGGTGCGGATGCTGGTTGCCACCGGCGGCCCCGCCCTCGTCAA
GGCGGTGCTGTCGACCGGCAAGAAGGCGATCGGCGCCGGCGCCGGCAATCCGCCCGTCGTGGTCG
ACGAAACCGCCAACATCGAAAAAGCCGCCTGCGACATCGTCAATGGCTGCAGCTTCGACAACAAC
ATAACCTGCACTGCCGAGAAGGAGATCATCGCAGTCGCCCAGATCGCCGATTACCTGATCTTCAA
CTTGAAGAAGAACGGCGCCTACGAGATCAAGGATCCGGCGGTTCTGCAGCAACTGCAGGACCTGG
TGCTGACCGCCAAGGGCGGGCCACAGACCAAATGCGTCGGCAAGAGTGCGGTGTGGCTGCTGTCG
CAGATCGGCATCAGCGTCGACGCCAGCATCAAGATCATCCTGATGGAAGTGCCGCGGGAACATCC
CTTCGTGCAGGAAGAACTGATGATGCCGATCCTGCCGCTGGTCCGGGTCGAAACCGTGGATGATG
CGATCGACCTCGCCATCGAGGTGGAGCACGACAATCGCCACACCGCGATCATGCATTCCACCGAT
GTGCGCAAGCTCACCAAGATGGCCAAGCTGATCCAGACCACGATCTTCGTGAAGAACGGCCCGTC
TTATGCGGGGCACGGCGCCGGCGGCGAGGGATATTCCACCTTCACCATCGCGGGTCCCACGGGCG

AGGGCCTGACCTCGGCGAAGTCGTTCGCGCGCCGCCGCAAATGCGTGATGGTGGAAGCGCTCAAT ATCCGTTGA

>GCR AA

MAHHHHHHVGTNDANIADVVTKVLGEYGAPGAVSVAALTAKSPDGKSNSSADADVV ARMVAKAIRDHAGTAQPSGNAATSSAAVSDGVFETMDAAVEAAALAQQQYLLCSMS DRARFVQGIRDVILNQDTLEKMSRMAVEETGMGNYEHKLIKNRLAGEKTPGIEDLTTD AFSGDNGLTLVEYSPFGVIGAITPTTNPTETIVCNSIGMLAAGNSVVFSPHGRARQVSL LLVRLINQKLAALGAPENLVVTVEKPSRENTLAMMAHPKVRMLVATGGPALVKAVLS TGKKAIGAGAGNPPVVVDETANIEKAACDIVNGCSFDNNITCTAEKEIIAVAQIADYLIF NLKKNGAYEIKDPAVLQQLQDLVLTAKGGPQTKCVGKSAVWLLSQIGISVDASIKIILM EVPREHPFVQEELMMPILPLVRVETVDDAIDLAIEVEHDNRHTAIMHSTDVRKLTKMAK LIQTTIFVKNGPSYAGHGAGGEGYSTFTIAGPTGEGLTSAKSFARRRKCVMVEALNIR

Supplemental References

1. Reger AS, Carney JM, Gulick AM (2007) Biochemical and crystallographic analysis of substrate binding and conformational changes in acetyl-CoA synthetase. *Biochemistry* 46(22):6536–6546.
2. Kabsch W (2010) Xds. *Acta Crystallogr Sect D Biol Crystallogr* 66(2):125–132.
3. Zarzycki J, Sutter M, Cortina NS, Erb TJ, Kerfeld CA (2017) In vitro reconstitution of the core chemistry of a glycyl radical enzyme- Associated bacterial microcompartment. *Sci Rep* 7:42757.
4. Adams PD, et al. (2010) PHENIX: a comprehensive Python-based system for macromolecular structure solution. *Acta Crystallogr Sect D Biol Crystallogr* 66(2):213–221.
5. Emsley P, Cowtan K (2004) Coot: model-building tools for molecular graphics. *Acta Crystallogr Sect D Biol Crystallogr* 60(12):2126–2132.
